# Bioinformatic Identification and Experimental Validation of a Prognostic Transcriptional Signature Derived from Asparagine Metabolism-Related Genes in Breast Cancer

**DOI:** 10.3390/biology15161441

**Published:** 2026-08-21

**Authors:** Tianyang Liu, Guijuan Zhang, Jialin Li, Xianxin Yan, Min Ma

**Affiliations:** 1 School of Traditional Chinese Medicine, State Key Laboratory of Bioactive Molecules and Druggability Assessment, Guangdong Basic Research Center of Excellence for Natural Bioactive Molecules and Discovery of Innovative Drugs, Jinan University, Guangzhou 510632, China; lty0505@stu2022.jnu.edu.cn; 2 School of Nursing, Jinan University, Guangzhou 510632, China; zhanggj@jnu.edu.cn; 3 The Oncology Department, The First Affiliated Hospital of Jinan University, Guangzhou 510630, China; lijialin@stu2023.jnu.edu.cn

**Keywords:** breast cancer, asparagine metabolism-related genes, machine learning, prognostic signature, experimental validation

## Abstract

Breast cancer (BRCA) possesses prominent molecular heterogeneity, where aberrant expression of genes annotated to asparagine metabolism networks drives malignant progression and therapeutic resistance. However, systematic construction of prognostic signatures from a holistic asparagine metabolic pathway perspective remains scarce, limiting the clinical translation of metabolic insights into prognostic tools. We integrated TCGA and GEO BRCA transcriptomic datasets to screen asparagine metabolism-related differentially expressed genes and build a prognostic model. Six biomarkers, SLC35A2, SRD5A2, NT5E, CEL, IFNG and CNR1, were selected via univariate Cox, LASSO and multivariate Cox regression. SRD5A2 and IFNG were enriched in low-risk patients, while the other four genes were upregulated in high-risk subgroups. This signature reliably stratifies patient prognosis, with risk scores correlating strongly with pathway activity, immune infiltration, immune checkpoints, mutation landscapes and drug responsiveness. Bioinformatic results were validated via TCGA cohort analysis, in vitro cellular assays and Western blot. Two in vivo models were established: 4T1 xenografts in 6-week-old BALB/c mice and DMBA/hormone-induced spontaneous breast tumors in 8-week-old SD rats. Tumors were generated by cell injection or DMBA gavage plus cyclic hormone treatment, and tissue sections were processed for immunohistochemistry. Consistent differential expression of the six core genes was validated across all in vitro and in vivo systems. In conclusion, this asparagine metabolism-associated signature offers candidate biomarkers for personalized prognosis and provides preclinical evidence for metabolism-targeted BRCA therapy.

## Simple Summary

Breast cancer remains a major threat to women worldwide, and patients often show different outcomes because tumors vary greatly in biology and treatment response. This study focused on genes from a broad asparagine metabolism-related gene set annotated by GeneCards. By combining public transcriptome datasets with statistical modeling and experimental validation, we identified six genes that can help distinguish patients with different prognostic risks. The model also reflected differences in immune cell infiltration, treatment sensitivity, and gene expression patterns across breast cancer samples, cell lines, and animal models. These findings may provide useful biomarkers for prognosis evaluation, rather than providing direct preclinical evidence for asparagine metabolism-targeted therapeutic strategies in breast cancer.

## 1. Introduction

Breast cancer (BRCA) represents the most prevalent malignant tumor in females worldwide and ranks among the leading causes of cancer-associated mortality globally [1]. Its global incidence continues to rise steadily at an annual rate of approximately 0.5% [2,3]. In 2020, BRCA surpassed lung cancer to become the most frequently diagnosed human malignancy and the fifth leading cause of cancer-related deaths, imposing a substantial socioeconomic burden on global public health systems [1]. The 2025 United States cancer statistics predicted 316,695 new BRCA cases and 42,170 BRCA-attributable deaths annually, establishing BRCA as the primary tumor-related lethal disease in women aged 20–49 years [4]. Current mainstream clinical therapeutic strategies for BRCA include surgical resection, chemotherapy, endocrine therapy, targeted immunotherapy (e.g., trastuzumab), and radiotherapy. Individualized treatment regimens are routinely formulated based on comprehensive evaluation of tumor stage, pathological typing, and molecular phenotypes of individual patients [5]. Despite remarkable advancements in modern therapeutics that have substantially improved the overall prognosis of BRCA patients, distant metastasis still occurs in 20–30% of patients after standardized curative treatment, accounting for nearly 90% of BRCA-related mortalities. Given its sophisticated molecular heterogeneity and complex pathogenic mechanisms, BRCA severely restricts the accuracy of tumor classification, standardization of clinical treatment, and precision of individualized prognostic evaluation [6]. Accordingly, precise identification of high-risk patient subgroups, reliable screening of robust prognostic biomarkers, exploration of novel therapeutic targets, and optimization of individualized treatment strategies are urgently required to improve long-term survival outcomes for BRCA patients. Breast cancer molecular heterogeneity hinders accurate identification of high-risk patients. Aberrant asparagine metabolism drives tumour proliferation, metastasis and drug resistance [7,8]. Identifying pathway-based prognostic biomarkers and building a multi-gene model helps achieve precise stratification and individualized therapy.

Asparagine, a conditionally essential amino acid, serves as a critical mediator of transmembrane amino acid exchange, maintains intracellular amino acid homeostasis, and sustains tumor anabolic metabolism, thereby facilitating the aberrant proliferation and progression of malignant cells. Accumulating evidence has identified asparagine metabolism as a promising therapeutic target for multiple cancers [9]. Asparagine synthetase (ASNS), a ubiquitously expressed enzyme in mammalian cells, catalyzes the de novo synthesis of asparagine using aspartate as the substrate and glutamine as the amino donor [10]. Elevated ASNS expression has been validated as an independent adverse prognostic biomarker in a wide spectrum of solid tumors [11]. Moreover, the successful clinical application of L-asparaginase in treating acute lymphoblastic leukemia further demonstrates the enormous translational potential of targeting asparagine metabolic pathways for cancer therapy [9]. Current mechanistic studies have clarified two predominant patterns by which solid tumors exploit asparagine to support malignant progression: active uptake of extracellular asparagine to fuel tumor growth, and adaptive utilization of asparagine as an alternative nutrient to sustain cell survival under glutamine deprivation [12]. In BRCA, asparagine not only supports the proliferation of breast cancer epithelial cells under glutamine-deficient conditions but also actively modulates tumor metastatic cascades [11].

Mechanistically, ASNS-driven asparagine accumulation promotes breast cancer lung metastasis [7]. SLC38A3 modulates glutamine, glutamate and aspartate levels to regulate tumour invasiveness [13]. The inhibitor CCT196969 suppresses triple-negative breast cancer by inhibiting ASNS-dependent asparagine synthesis and mTORC signalling [14]. Nevertheless, most studies focus on individual metabolic enzymes. Few reports construct breast cancer prognostic signatures by systematically screening hub genes from asparagine metabolism-related genes, and the links between these signatures, immune microenvironment and drug response remain poorly understood. Mounting evidence demonstrates that remodelling of the multi-dimensional asparagine metabolism regulatory network fuels breast cancer progression and prognostic heterogeneity. This paradigm contains three core aspects: (1) Breast cancer cells achieve asparagine metabolic reprogramming via ASNS-dependent de novo synthesis, extracellular asparagine uptake and compensatory pathways; (2) This metabolic rewiring coordinates substrate supply, energy metabolism and immune regulation instead of relying on independent enzymatic reactions, and hub gene combinations possess superior predictive value over single metabolic enzyme markers; (3) The global state of this network remodels the tumour microenvironment and modulates drug response, enabling prognostic stratification and supporting metabolism-targeted combination therapies. Accordingly, we screened key transcriptional nodes within asparagine metabolism-associated genes and constructed a multi-gene prognostic model, followed by analyses of its clinical and biological correlations with immune characteristics and drug sensitivity. Importantly, this transcriptome-derived signature cannot directly represent real asparagine metabolic activity.

Existing studies have verified that asparagine metabolism drives breast cancer progression, yet few systematic multi-gene prognostic models have been developed based on the full asparagine metabolic pathway. The clinical value of pathway signatures for prognosis prediction, immune microenvironment analysis and drug sensitivity evaluation remains unclear. To address this gap, we integrated transcriptomic data from TCGA and multiple GEO breast cancer cohorts. Asparagine metabolism-related genes were intersected with breast cancer differentially expressed genes, and survival analyses identified core prognostic genes. We characterized these genes and validated predictions preliminarily. We constructed and externally validated a novel multi-gene risk model, explored its links with clinical features, immune infiltration and chemotherapeutic response, and verified hub gene function in vitro. This signature may facilitate precise risk stratification and supply biomarkers for individualized therapy.

## 2. Materials and Methods

### 2.1. Data Source

RNA-seq data of the TCGA-BRCA cohort were downloaded from the UCSC Genome Browser (https://genome.ucsc.edu/, accessed on 21 April 2025), including STAR-generated raw count matrices and FPKM matrices. Raw counts were adopted for differential expression analysis via DESeq2, while FPKM values were used for gene expression comparison, survival analysis and prognostic signature construction. Ensembl gene IDs were converted to gene symbols based on the GENCODE v36 annotation file, and only protein-coding genes were retained. For multiple Ensembl entries mapped to a single gene symbol, the entry with the highest average expression across all samples was reserved to guarantee one expression profile per gene symbol. Sample types were classified according to the 14th–15th digits of TCGA sample barcodes: codes 01–09 represented tumor tissues, and codes ≥10 indicated normal tissues. Expression matrices were matched with clinical survival data by sample ID, and specimens lacking essential survival information were excluded. After filtering, 1091 breast tumor samples and 112 normal tissue samples were finally enrolled in the TCGA-BRCA cohort. The standardized gene-level expression matrix of the external validation dataset GSE96058 was retrieved from the Gene Expression Omnibus (GEO, https://www.ncbi.nlm.nih.gov/geo/, accessed on 21 April 2025). Since the matrix had been fully preprocessed and transformed by the original study, no additional cross-sample normalization or log_2_ transformation was performed in this work. The matrix was already aggregated at the gene level rather than the probe level, so probe-to-gene symbol conversion and multi-probe aggregation were unnecessary. Genes containing missing expression values were removed after quality inspection, and no duplicated gene symbols were detected, requiring no further deduplication. Clinical prognostic information of GSE96058 was extracted from phenotype data corresponding to platform GPL18573 [15]. The overall survival event status and overall survival days were extracted for subsequent analysis. Samples with missing survival information or follow-up time ≤ 0 were discarded. Expression profiles and clinical data were intersected and sorted consistently by sample ID, and 340 breast cancer patients with complete expression and survival records were retained for external validation. Furthermore, 1295 asparagine metabolism-related genes (AMRGs) were obtained with a relevance score greater than 6 via the GeneCards database. This threshold was preset during data retrieval and was only used to preliminarily define the candidate gene pool for screening.

### 2.2. Attainment and Functional Exploration for Candidate Genes (CGs)

DEGs between the BRCA and control groups from the TCGA-BRCA were obtained utilizing the DESeq2 package (v. 1.42.0) [16]. The thresholds were set at adjusted (adj). *p* < 0.05 and |log_2_FC| > 1. The up- and down-regulated top 10 DEGs were labeled by ascending *p*-value order. In order to obtain the differential genes associated with AMRGs in BRCA, DEGs were additionally overlapped with AMRGs to obtain CGs, which were further displayed in a Venn diagram using the ggvenn package (v. 1.7.3) [17]. Then, CGs were interpreted by Gene Ontology (GO) and Kyoto Encyclopedia of Genes and Genomes (KEGG) analyses (adj. *p* < 0.05) using the Cluster profiler package (v. 4.12.6) [16]. The top 10 pathways sorted by ascending order of adj. *p* were selected from the above results.

To explore protein interactions of CGs, a protein-protein interaction (PPI) network was constructed (confidence scores > 0.4) via the STRING database.

### 2.3. Prognostic Genes Screening

In order to explore the prognostic genes related to BRCA, a univariate Cox analysis was applied on CGs using the survival package (v. 3.7.0) (https://github.com/therneau/survival accessed on 21 April 2025), and candidate prognostic genes (CPGs) were obtained. To further confirm the validity and reliability of the CPGs, the Schoenfeld residual method was employed for the proportional hazards (PH) assumption test (*p* > 0.05). Next, CPGs were screened by the Least absolute shrinkage and selection operator (LASSO) regression through the glmnet package (v. 4.1.8) [18]. Genes with higher relevance to BRCA were retained with reduced coefficients, while those with lower significance were compressed to zero. After 10-fold cross-validation, the graphs of LASSO regression gene coefficients and the error plots of cross-validation were obtained. Further screening was performed by multivariate Cox analysis and PH assumption test.

### 2.4. Construction, Assessment, and Validation of Risk Model

Using the prognostic risk model generated from the final prognostic genes, the BRCA samples with survival information in the TCGA-BRCA were used to calculate the BRCA patients’ risk scores. The Random Survival Forest (RSF) model was constructed solely based on the TCGA-BRCA training cohort with ntree = 1000 and mtry = 3 set as hyperparameters. The ensemble cumulative hazard output by the model was defined as the continuous risk score, and no fixed linear coefficients similar to those from Cox regression were generated.

RiskScore_i_ = ∑_k=1_^K^ Ĥ_i_(t_k_)

where Ĥ_i_(t_k_) denotes the ensemble cumulative hazard function of patient at event time t_k_. A higher risk score corresponds to a higher predicted risk of death. As the RSF model is a nonparametric tree ensemble algorithm, it does not yield a fixed linear coefficient formula like the Cox regression model. And the BRCA patients were categorized into HRG and LRG based upon the optimal cutoff value of the risk score (res.cut), and a corresponding risk curve was plotted in ascending order of patient risk scores. Further assessment of survival differences between groups was performed by plotting Kaplan–Meier (K-M) survival twist. The significance of the survival difference was compared by applying the Log-rank test (*p* < 0.05). Subsequently, corresponding ROC curves were obtained by the survivalROC package (v. 1.18.0) [19] at 1, 3, and 5 years. To further assess the changes in prognostic gene expression levels among different samples of the two groups, the pheatmap package (v. 1.0.12) was used to show prognostic gene expression. Finally, the above analyses were conducted in the validation dataset GSE96058. Prognostic genes were identified and the risk model were constructed based on the training cohort, and GSE96058 was utilized solely as an external validation set to assess the model’s generalizability across distinct detection platforms and patient populations.

### 2.5. Relationship Between Risk Scores and Clinicopathologic Characteristics

The Wilcoxon test was utilized to contrast risk score differences across clinical characteristics (such as age, stage, etc.) of BRCA patients and evaluate the disparities in the proportion of patients between the two groups according to the data in the TCGA-BRCA, with *p* < 0.05 as the threshold. Lastly, the correlations between risk groups and different clinical characteristics were analyzed.

### 2.6. Gene Set Enrichment Analysis (GSEA)

In the TCGA-BRCA, the DESeq2 package was utilized to judge the difference in biological process between the two groups. The log_2_FC value was calculated and ranked in descending order. In addition, the ClusterProfiler package [20] was applied to execute GSEA on the two groups of the TCGA-BRCA (*p* < 0.05, |NES| > 1, and false discovery rate (FDR) < 0.25). The reference gene collections were shown in Table S1.

### 2.7. Immune Infiltration Analysis

Between two groups of the training set, the infiltration ratios of 22 immune cell subsets in each sample were evaluated via the CIBERSORT package (v. 0.1.0) [21]. The Wilcoxon test was used to evaluate the disparity in the profusion of these immune cell subsets between the two groups. Finally, the connection between prognostic genes and significantly differential immune cells (SDICs), and the correlation between SDICs was calculated by Spearman analysis (|correlation| > 0.3, *p* < 0.05) through the Hmisc package (v. 5.1.3) [22]. In addition, group comparisons were performed for 22 immune cell types, and the Benjamini–Hochberg method was used for multiple testing correction with FDR < 0.05 as the threshold.

### 2.8. Tumor Purity Analysis and Immunization Checkpoint Variance Analysis

To further explore the differences in tumor scores in the two groups, the ESTIMATE method was used on all samples to evaluate the tumor tissue stromal score and immune cell infiltration score based on the ESTIMATE database (https://bioinformatics.mdanderson.org/estimate/ accessed on 21 April 2025). And the two scores were combined to calculate the tumor purity score. Finally, the Wilcoxon test was employed to evaluate the disparity in tumor pureness, ESTIMATE score, immune score, and stromal score in the two groups.

The expression of immune checkpoints was further evaluated through the Wilcoxon test based on BRCA patients with survival information in the TCGA-BRCA. And *p* < 0.05 represents a significant difference.

### 2.9. Drug Sensitivity Analysis

To investigate the medicinal effects of chemotherapeutic drugs in BRCA patients, chemotherapeutic drugs and their half-maximal inhibitory concentration (IC_50_) values for BRCA were acquired from the Genomics of Drug Sensitivity in Cancer (GDSC) database. In addition, the oncoPredict package (v. 1.2) [23] was used to evaluate the IC_50_ value of each chemotherapeutic drug, and the Wilcoxon test was adopted to show the discrepancy in IC_50_ values of the chemotherapeutic drugs between the two groups. DEGs were obtained, and the top 10 DEGs sorted by ascending *p* value order were shown.

### 2.10. Tumor Mutation Analysis and Validation of Prognostic Gene Expression Levels

To compare somatic mutation differences between the two groups, somatic mutation data for each BRCA sample in the TCGA-BRCA (n = 1002) were obtained. The Maftools package (v. 2.14.0) [24] was used for somatic mutation analysis, and the top 20 genes were exhibited in descending mutation frequency order. Next, the DESeq2 package was adopted to assess the disparity of mutated genes between the two groups (adj. *p* value < 0.05, |log_2_FC| > 1), obtaining differential mutation genes (DMGs). Then, the survival package was applied to conduct univariate Cox analysis on DMG, and key mutated genes (KMGs) were identified for subsequent analysis. Next, to explore the impact of KMGs on proteins and the distribution of mutation sites, lollipop was used to visualize the mutation sites of KMGs. The top three key mutation sites were selected, and the results were presented in a lollipop plot. To explore the mutation status of prognostic genes, mutation analysis was performed according to BRCA patient data from the CBio Cancer Genomics Portal database.

To reveal the expression levels of the prognostic genes in the TCGA-BRCA, a *t*-test was applied to all samples in the training set to assess expression discrepancies in the two groups (Tumor vs. Normal).

### 2.11. Cell Culture and RT-qPCR Assay

Human breast cancer cell lines, including MCF-7 (Luminal subtype, ER+/PR+/HER2−, RRID:CVCL_0031), SK-BR-3 (HER2-overexpressing subtype, ER−/PR−/HER2+, RRID:CVCL_0033), MDA-MB-231 (claudin-low triple-negative breast cancer, TNBC, ER−/PR−/HER2−,RRID:CVCL_0062) and SUM-159PT (basal-like TNBC, ER−/PR−/HER2−, RRID:CVCL_5423), were preserved in The Fang Zheng laboratory of Jinan University. The human normal mammary epithelial cell line MCF-10A (RRID:CVCL_0598) was purchased from Procell Life Science & Technology Co., Ltd., Wuhan, Hubei, China, and cultured in the proprietary medium specially formulated for MCF-10A cells supplied by the same vendor. SK-BR-3 cells were maintained in complete McCoy’s 5A medium (GIBCO, Thermo Fisher Scientific, Waltham, MA, USA) supplemented with 10% fetal bovine serum (FBS). MCF-7, MDA-MB-231, and SUM-159PT cells were cultured in high-glucose DMEM (GIBCO, Thermo Fisher Scientific, Waltham, MA, USA) containing 10% FBS. All culture media were supplemented with 1% penicillin–streptomycin solution to prevent bacterial contamination. All cell lines were cultivated in a humidified incubator at 37 °C with 5% CO_2_ following standard in vitro cell culture protocols.

Total cellular RNA was extracted using TRIzol reagent (Life Technologies, Carlsbad, CA, USA) after cells were rinsed with ice-cold phosphate-buffered saline (PBS). RNA concentration and purity were determined using a NanoDrop spectrophotometer, and samples with an OD260/OD280 ratio > 2.0 were considered eligible for subsequent experiments.

Total RNA was reverse-transcribed into cDNA using the Evo M-MLV RT Mix Kit with gDNA Clean (Accurate Biology, Changsha, Hunan, China) to eliminate genomic DNA contamination. Quantitative real-time PCR was performed using the HS SYBR Green Premix Pro Taq qPCR Kit (Accurate Biology, Changsha, Hunan, China) on a CFX Real-Time PCR System. Briefly, residual genomic DNA was removed at 42 °C for 2 min, followed by reverse transcription at 55 °C for 15 min and enzyme inactivation at 85 °C for 5 s. The qPCR reaction system was prepared strictly according to the manufacturer’s instructions. Each sample was analyzed in triplicate, and all experiments were repeated three times independently. β-actin was used as the internal reference gene, and the relative mRNA expression levels were calculated using the 2^−ΔΔCt^ method. All primer sequences were designed and verified using NCBI Primer-BLAST (v.2.5.0) to ensure specific amplification, as listed in Table 1.

### 2.12. Western Blot Analysis

Total cellular protein was extracted from cultured cells using RIPA lysis buffer containing protease inhibitor cocktail (Epizyme, Shanghai, China). After quantitative detection, equal amounts of protein were separated by SDS-PAGE and electrotransferred onto PVDF membranes. The membranes were blocked with 5% non-fat milk at room temperature and incubated overnight at 4 °C with specific primary antibodies. The primary antibodies used in this study included anti-SLC35A2 (1:1000, #13657-1-AP, Proteintech, Wuhan, China), anti-IFNG (1:1000, #TD6045, Abmart, Shanghai, China), anti-CNR1 (1:2000, #17978-1-AP, Proteintech, Wuhan, China), and anti-β-actin (1:10,000, #66009-1-Ig, Proteintech, Wuhan, China). On the following day, the membranes were incubated with corresponding HRP-conjugated secondary antibodies at room temperature, including HRP-conjugated Goat Anti-Rabbit IgG (H + L) (1:5000, #SA00001-2, Proteintech, Wuhan, China) and HRP-conjugated Goat Anti-Mouse IgG (H + L) (1:5000, #SA00001-1, Proteintech, Wuhan, China). Protein bands were visualized using ECL chemiluminescence reagent. The relative band intensity was quantified with ImageJ software (v.1.54f) and normalized to β-actin. All experiments were repeated in three independent biological replicates.

### 2.13. Establishment of a DMBA Combined with Estrogen-Progesterone-Induced Spontaneous Breast Cancer Model in SD Rats

To verify the clinical biological correlation between asparagine metabolism dysregulation and breast cancer malignant progression under physiological hormone regulation conditions, and to observe the in vivo expression characteristics of core metabolic genes during tumor spontaneous development, this spontaneous breast cancer model was constructed. All animal experiments were approved by the Animal Ethics Committee of Jinan University (Approval No. 20221108-01). Six-week-old virgin female specific-pathogen-free (SPF) SD rats weighing 180–200 g were purchased from Beijing HFK Bioscience Co., Ltd., Changping District, Beijing, China. Rats were randomly assigned to the normal control group and tumor model group (n = 6 per group). Rats in the model group received a single intragastric dose of DMBA dissolved in sesame oil (7 mg DMBA per rat), whereas control rats were administered an equal volume of pure sesame oil. Starting on day 2 after model induction, cyclic hormone intervention was performed for 12 consecutive cycles, with each cycle lasting 5 days. For each cycle, estradiol benzoate (0.5 mg/kg) was intramuscularly injected on days 1–3, progesterone (4 mg/kg) on day 4, and no treatment was applied on day 5. All rats were maintained under standard SPF housing conditions. At the end of the experiment, mammary gland tissues were harvested for subsequent immunohistochemical detection of core asparagine metabolic genes, so as to clarify their expression changes during endogenous breast cancer tumorigenesis.

### 2.14. 4T1 Xenograft Tumor Model and Paclitaxel Intervention in Nude Mice

The 4T1 xenograft tumor model with paclitaxel intervention was established to explore the regulatory effect of paclitaxel on asparagine metabolism in breast cancer and validate the metabolic mechanism underlying tumor drug response in vivo. The animal protocol for nude mouse experiments was approved by the Animal Ethics Committee of Jinan University (IACUC-20250923-02). Six- to eight-week-old healthy female SPF BALB/c nude mice weighing 18–22 g were obtained from Guangdong Vital River Laboratory Animal Technology Co., Ltd., Foshan, Guangdong, China. and acclimatized under sterile housing conditions. A murine breast cancer xenograft model was established via subcutaneous injection of 4T1 cells. After stable tumor formation, mice were randomly divided into an untreated model group and a paclitaxel treatment group (n = 6 per group). Mice in the treatment group received intraperitoneal injection of paclitaxel (10 mg/kg) once every two days for 21 consecutive days, while the model group was injected with an equal volume of normal saline. Tumor tissues were isolated and fixed for immunohistochemical staining to compare the differences in core metabolic gene expression between the blank model group and paclitaxel intervention group, further confirming the in vivo regulatory relationship between chemotherapy drugs and asparagine metabolism.

### 2.15. Immunohistochemical Staining

Collected rat mammary gland tissues and mouse xenograft tumor tissues were fixed in 4% paraformaldehyde, embedded in paraffin, and serially sectioned. After routine dewaxing, rehydration, antigen retrieval, and blocking, tissue sections were incubated with primary antibodies against SLC35A2 and IFNG overnight at 4 °C, followed by incubation with HRP-labeled secondary antibodies. The positive staining signals were visualized using DAB chromogenic reagent, and nuclei were counterstained with hematoxylin. After dehydration and mounting, microscopic images were captured. The protein expression levels were quantitatively evaluated according to the staining intensity and positive cell proportion in each field.

### 2.16. Statistical Analysis

All bioinformatics analyses were performed using R software (version 4.3.3). Wilcoxon test was applied for two-group differential analysis. Univariate and multivariate Cox regression analyses were performed to screen prognostic genes, with *p* < 0.05 and HR ≠ 1 defined as statistically significant. The proportional hazards (PH) assumption was verified to ensure model applicability. For in vitro cellular experiments, statistical analysis and graph plotting were conducted using GraphPad Prism 10.0. Independent samples *t*-test was used for two-group comparisons, and one-way ANOVA was used for multi-group comparisons. All quantitative data are presented as mean ± standard deviation (SD). Each experiment was repeated at least three times independently. *p* < 0.05 was considered statistically significant, while *p* ≥ 0.05 was defined as no significant difference (ns). All enrichment and immune infiltration analyses in this study were bioinformatic correlation analyses and were not equivalent to biological validation via in vitro or in vivo experiments, thus overinterpretation of mechanistic conclusions was avoided.

## 3. Results

### 3.1. Functional Exploration of 334 CGs

**Figure 1:**
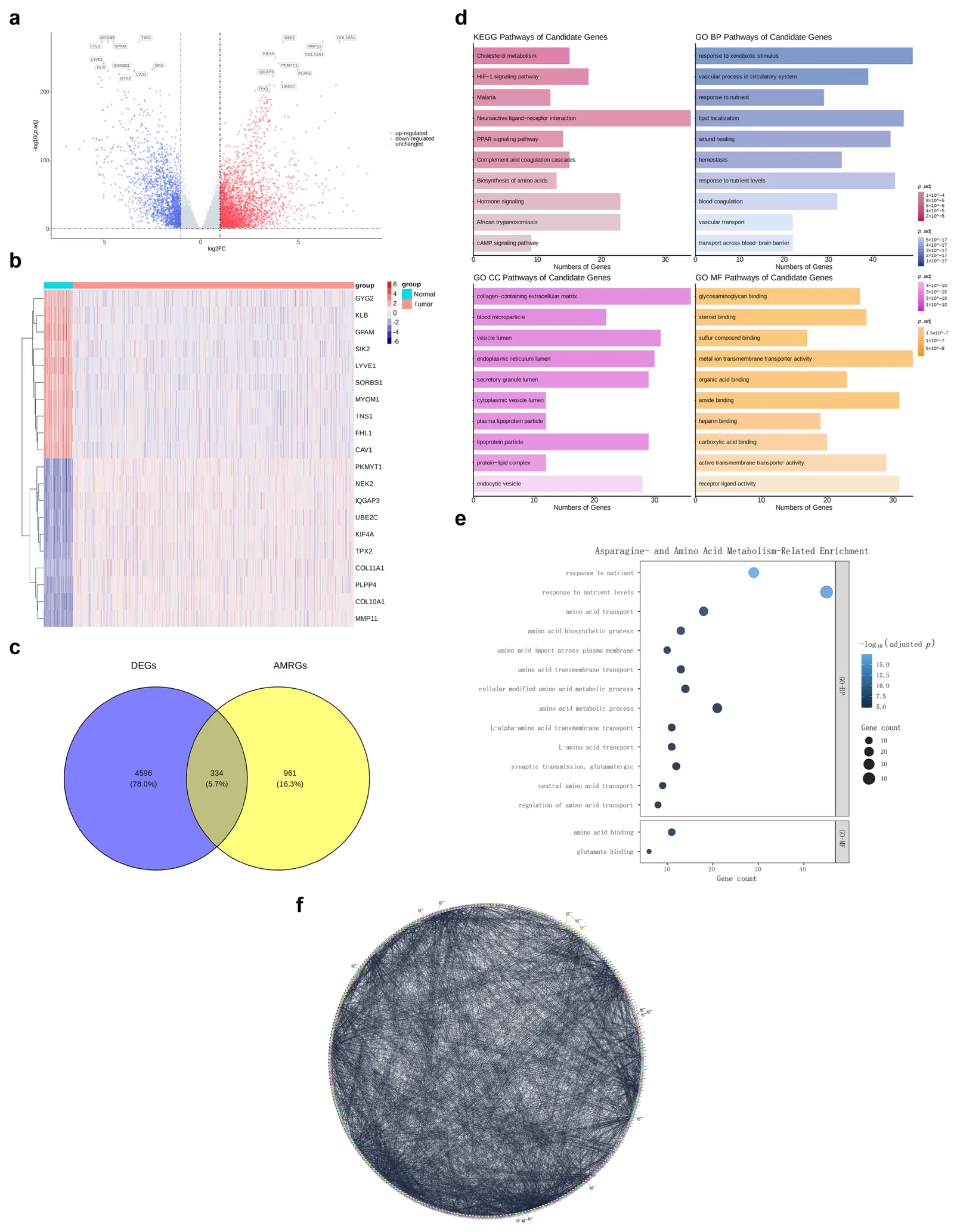
Differential expression analysis of BRCA samples versus normal samples from the TCGA-BRCA dataset. (**a**) Volcano plot of differentially expressed genes between normal samples and BRCA samples. Note: Genes in the upper right corner represented upregulated differentially expressed genes (marked in red), genes in the upper left corner represented downregulated differentially expressed genes (marked in blue), and the remaining genes represented genes without statistically significant differences (marked in gray) The vertical dashed lines correspond to |log_2_FC| = 1, and the horizontal dashed line indicates adj. *p* = 0.05. (**b**) Heatmap of the top 10 differentially expressed genes in both up- and down-regulated groups. Note: Red indicated highly expressed genes, and blue indicated lowly expressed genes. (**c**) candidate gene screening Venn diagram. (**d**,**e**) GO and KEGG enrichment analysis of candidate genes. Note: Darker colors in the figure indicated higher significance of the corresponding pathways. (**f**) PPI network of candidate genes. Note: Dots represented proteins, and lines represented pairwise protein-protein interactions.

Altogether, 4930 DEGs were acquired via differential analysis with adj. *p* < 0.05, |log_2_FC| > 1, including 3009 up- and 1921 down-regulated genes. Figure 1a,b were constructed to display DEGs, and the top 10 upregulated and top 10 downregulated differentially expressed genes were labeled according to *p*-value sorting. The intersection of 4930 DEGs and 1295 AMRGs yielded 334 CGs (Figure 1c). Furthermore, in the GO enrichment analysis of the CGs, a total of 2011 results were obtained (Figure 1d). And the KEGG enrichment analysis yielded a total of 61 results (Figure 1d). Specifically, the top ten results in BP, CC, and MF were related to vascular function, protein particles, and compound binding, respectively. Meanwhile, the top ten terms in KEGG were mainly concentrated in signal transduction. Furthermore, we focused on entries related to amino acid metabolism, amino acid transport, nutrient response and glutamate-associated pathways for presentation. The results showed that the candidate genes were significantly enriched in multiple amino acid-related biological processes, including amino acid metabolic process, amino acid biosynthetic process, amino acid transport, and amino acid transmembrane transport. Significant enrichments of amino acid binding and glutamate binding were also observed at the molecular function level (FDR < 0.05) (Figure 1e). The PPI network illustrates the protein-level interactions among CGs with a total of 329 proteins and 3771 pairs of interacting proteins pairs in the network (Figure 1f).

### 3.2. Acquisition of Prognostic Genes SLC35A2, SRD5A2, NT5E, CEL, IFNG, and CNR1

**Figure 2:**
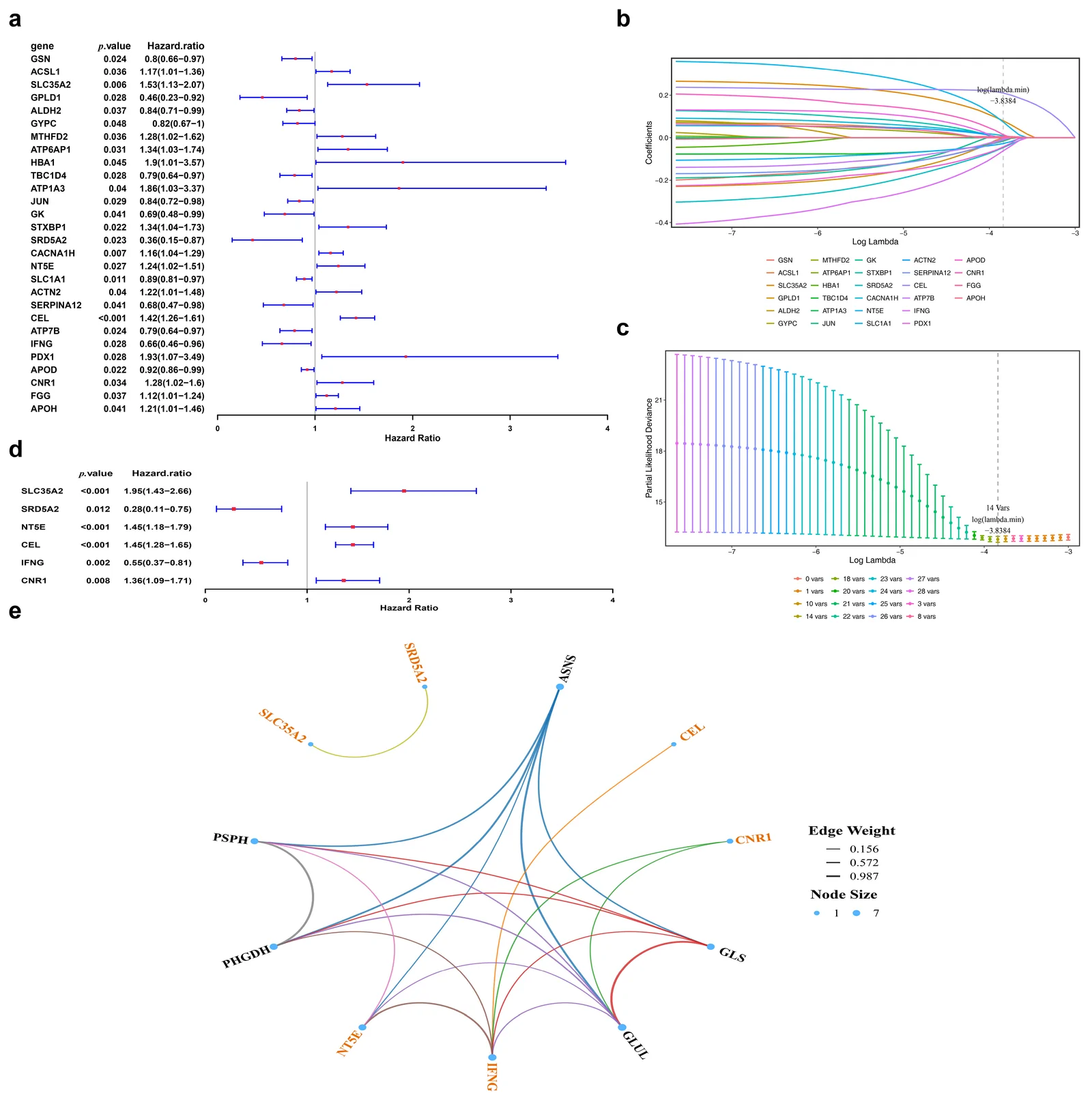
Acquisition of prognostic genes SLC35A2, SRD5A2, NT5E, CEL, IFNG, and CNR1. (**a**) Identify genes associated with BRCA prognosis through univariate Cox regression analysis. Note: The horizontal axis represented HRs. Red dots indicated HR values, and blue lines denoted confidence intervals. (**b**) Lasso regression gene coefficient plot. (**c**) Lasso regression cross-validation error plot. (**d**) Identify prognostic genes for BRCA through multivariate Cox analysis. Note: The horizontal axis represented HRs. Red dots indicated HR values, and blue lines denoted confidence intervals. (**e**) PPI network of prognostic genes and asparagine metabolism-related genes.

After performing univariate Cox regression analysis on 334 CGs, 28 genes associated with BRCA prognosis were identified. As shown in Figure 2a, 28 genes with HR ≠ 1 and *p* < 0.05 were screened out. Further PH assumption tests were conducted on these 28 genes, and all of which passed the tests, indicating reliable results (*p* > 0.05) (Table S2). In the LASSO regression analysis for eliminating redundant genes and avoiding model overfitting, 14 candidate genes were retained at the optimal λ value (log(lambda.min) = −3.8384) corresponding to the model’s minimum cross-validation error (Figure 2b,c, Table S2). Multivariate Cox analysis and PH assumption tests were performed on the 14 genes and ultimately identified 6 prognostic genes: SLC35A2 (HR = 1.95 95% CI 1.43–2.66), SRD5A2 (HR = 0.28 95% CI 0.11–0.75, NT5E (HR = 1.45 95% CI 1.18–1.79), CEL (HR = 1.45 95% CI 1.28–1.65), IFNG (HR = 0.55 95% CI 0.37–0.81), and CNR1 (HR = 1.36 95% CI 1.09–1.71) (*p* < 0.05) (Table S3) (Figure 2d). Finally, the interaction relationships between these six prognostic genes and the core aspartate metabolism genes (ASNS, GLS, GLUL, PHGDH, and PSPH) were extracted from the PPI network. The network revealed that prognostic genes exhibited certain interactions with these core genes: ASNS was found to interact with NT5E; GLS interacted with IFNG; and GLUL showed interactions with NT5E, IFNG and CNR1 (Figure 2e).

### 3.3. Upregulation of SRD5A2 and IFNG and Downregulation of SLC35A2, NT5E, CEL and CNR1 in the LRG

**Figure 3:**
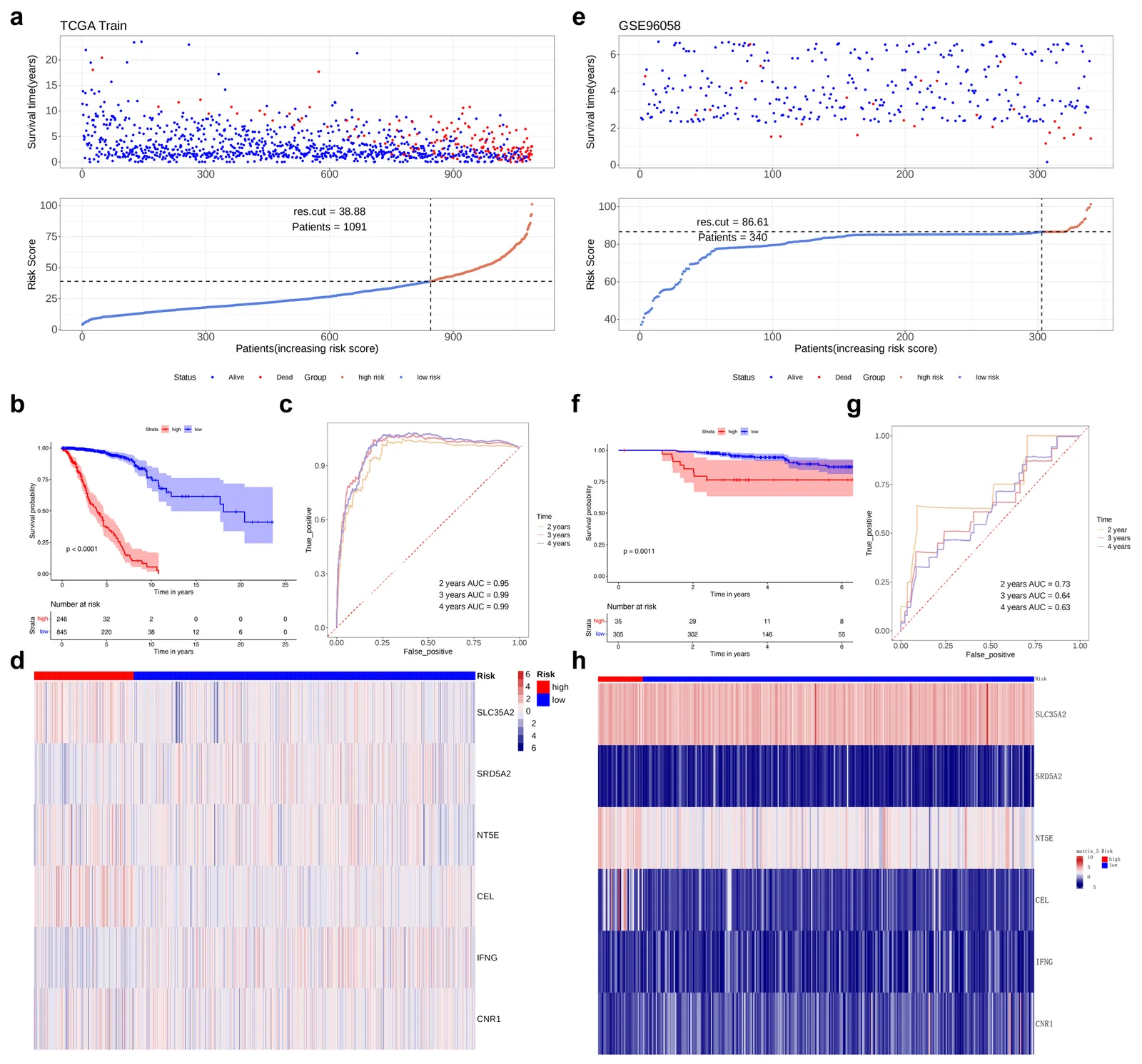
Establishment and evaluation of a prognostic model based on six prognostic genes. (**a**) Survival status distribution of patients (**top**) and risk score distribution of patients (**bottom**) in the TCGA-BRCA training set. The vertical dashed line represents the optimal cutoff value (res.cut = 38.88) for risk stratification; the horizontal dashed line corresponds to the cutoff risk score. (**b**) K-M survival curves for high- and low-risk groups of patients (TCGA-BRCA training set). (**c**) ROC curves for risk model prediction of patient survival (1, 3, 5 years) in the TCGA-BRCA training set. Note: AUC values represented the area under the curve. (**d**) Heatmap of prognostic gene expression for high- and low-risk groups of patients (TCGA-BRCA training set). Note: Red represented high gene expression and blue represented low gene expression in the heatmap. (**e**) Validation set GSE96058, (**top**): Patient survival status distribution plot; (**bottom**): Patient risk score distribution plot. The vertical dashed line represents the optimal cutoff value (res.cut = 86.61) for risk stratification; the horizontal dashed line corresponds to the cutoff risk score. (**f**) K-M survival curves for high- and low-risk group patients in the TCGA-BRCA validation set GSE96058. (**g**) TCGA-BRCA Validation set GSE96058, ROC curve for risk model predicting patient survival (1, 3, 5 years). Note: AUC values represented the area under the curve. (**h**) TCGA-BRCA Validation set GSE96058, heatmap of prognostic gene expression in high- and low-risk groups). Note: Red represented high gene expression and blue represented low gene expression in the heatmap.

First, BRCA patients with survival information were classified into HRG (high-risk group, n = 246) and LRG (low-risk group, n = 845) with the optimal risk score cutoff (res.cut = 38.88) (Figure 3a) (Table S4). Kaplan–Meier survival analysis further verified that patients in the HRG exhibited markedly poorer overall survival rates compared with the LRG (Figure 3b). To quantitatively evaluate the predictive performance of the six-gene signature, time-dependent ROC curves at 2-, 3-, and 4-year follow-up endpoints were plotted. In the training cohort, the AUC values (95% CI) for 2-, 3-, and 4-year survival were 0.949 (0.902–0.988), 0.987 (0.945–0.999), and 0.991 (0.942–1.000), respectively. The results showed that all AUC values exceeded 0.6, which indicated that this six-gene signature possessed reliable discrimination ability to predict long-term survival status of BRCA patients (Figure 3c). The prognostic gene expression profiles revealed that SRD5A2 and IFNG were upregulated in the LRG. Conversely, SLC35A2, NT5E, CEL, and CNR1 were downregulated in the same group. (Figure 3d).

Similarly, HRG (n = 35) and LRG (n = 305) (res.cut = 86.61) were derived from the GSE96058 dataset (Figure 3e). K-M curve analysis confirmed pronounced survival difference between groups (*p* = 0.028) (Figure 3f). The 2-, 3-, and 4-year AUCs all surpassed 0.6 (Figure 3g), validating the model’s efficacy. In the validation cohort, the corresponding AUC values (95% CI) were 0.731 (0.577–0.967), 0.640 (0.466–0.776), and 0.628 (0.526–0.737) Consistent with the training set, the heatmap showed SRD5A2/IFNG overexpression and SLC35A2/NT5E/CEL/CNR1 underexpression in LRG (Figure 3h).

### 3.4. HRG and LRG Patients Had Significant Disparities in Clinical Stage, T Stage, and N Stage

**Figure 4:**
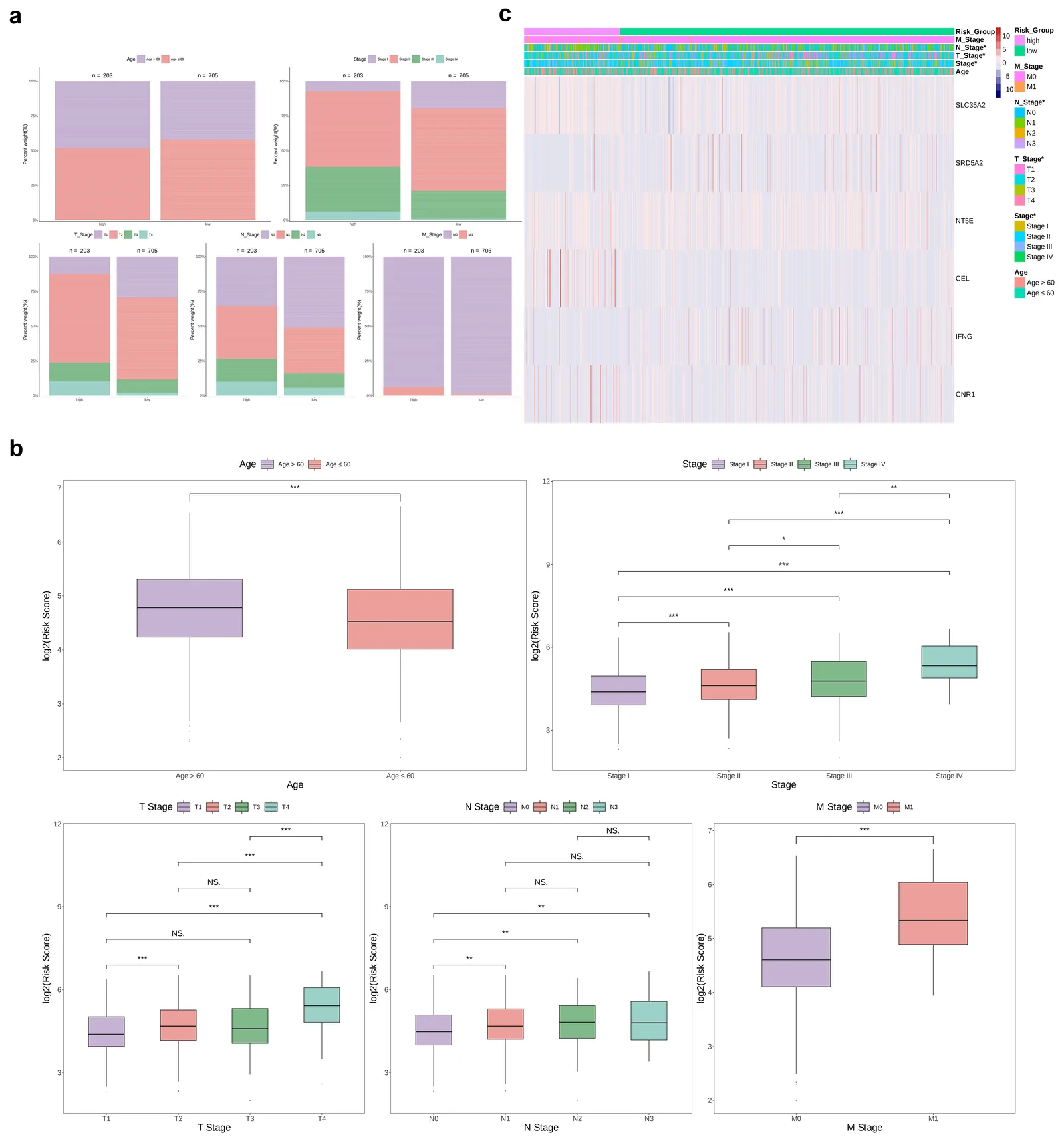
The relationship between risk scores and clinical pathological characteristics. (**a**) Stacked bar chart of clinical characteristic groupings. Note: Each bar represented the number of patients in the high-risk and low-risk groups, and different colors corresponded to distinct subtypes of clinical characteristics. (**b**) Analysis of differences in clinical characteristics based on risk scores. (**c**) Heatmap of clinical characteristics based on prognostic genes.* *p* < 0.05, ** *p* < 0.01, *** *p* < 0.001.

As shown in Figure 4a, clinical feature risk score analysis revealed significant differences in the proportions of Clinical Stage, T Stage, N Stage, and M Stage classifications between the two groups (*p* < 0.05). Meanwhile, significant disparities in risk scores for Clinical Stage, T Stage, and N Stage classifications were also identified (Figure 4b). In addition, substantial correlations were revealed among the risk groups and all classifications except for M Stage (*p* < 0.05). For example, SLC35A2 expression was significantly upregulated in N2 and T2 Stages (Figure 4c).

### 3.5. Chromatin, Chromosome, and Protein Functions Were Identified as Differentially Enriched Pathways

**Figure 5:**
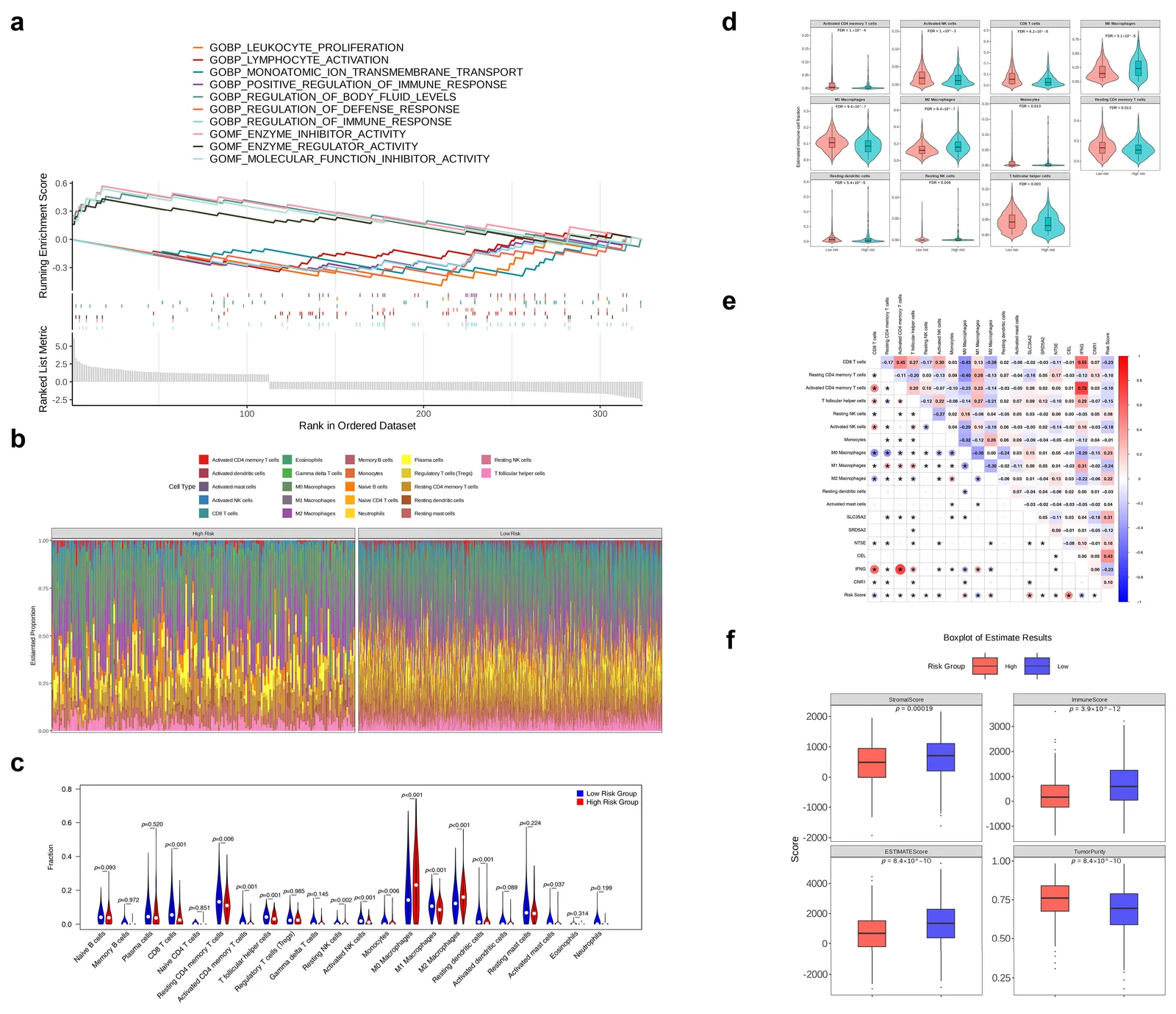
TCGA-BRCA GSEA enrichment analysis and immune infiltration base high and low risk groups. (**a**) GSEA enrichment analysis KEGG results based on high- and low-risk groups. Colored curves correspond to the running enrichment score of individual GO gene sets. Vertical tick marks indicate the positions of genes belonging to each gene set within the ranked gene list. The bottom panel displays the ranked list metric for all genes sorted according to expression variation. (**b**) Overlay histogram showing the proportion of immune cells in the high-risk and low-risk groups. (**c**) Violin plots showing the comparative differences in 22 immune cell types between the high-risk and low-risk groups. (**d**) Group comparisons were performed for 22 immune cell types, and the Benjamini–Hochberg method was used for multiple testing correction with FDR < 0.05 as the threshold. (**e**) Correlation analysis between prognostic genes and differentially expressed immune cells, as well as correlation analysis among differentially expressed immune cells. * *p* < 0.05. (**f**) Box plots showing differences in stromal score, immune score, ESTIMATE score, and Tumor Purity between high-risk and low-risk groups, * *p* < 0.05, ** *p* < 0.01, *** *p* < 0.001, **** *p* < 0.0001.

GSEA enrichment analysis showed that 57 differentially enriched pathways were identified between the two groups of BRCA in the TCGA-BRCA (*p* < 0.05, FDR *<* 0.25, and |NES| > 1) (Table S5). The top ten statistically enriched pathways were molecular function inhibitor activity, enzyme inhibitor activity, regulation of immune response, enzyme regulator activity, lymphocyte activation, monoatomic ion transmembrane transport, leukocyte proliferation, etc. These pathways are mainly involved in enzyme activity regulation, immune cell activation and proliferation, ion transmembrane transport and homeostasis maintenance of body fluid (Figure 5a).

### 3.6. Twelve Differentially Expressed Immune Cells Were Inversely Correlated with Prognostic Genes

Based on CIBERSORT predictions, the percentage of 22 types of immune cell infiltration showed that M0 macrophages accounted for a larger proportion in the HRG. While in LRG, the proportions of various cell types were more balanced, except for activated CD4 memory T cells, which accounted for a lower proportion (Figure 5b). These immune cell proportions represent computational estimates derived from transcriptomic deconvolution and should be interpreted as in silico predictions rather than direct experimental measurements.

A Wilcoxon test identified 11 immune cell types with notable discrepancies in the two groups, including M0 Macrophages and Monocytes among others (*p* < 0.05) (Figure 5c). Group comparisons were performed for 22 immune cell types, and the Benjamini-Hochberg method was used for multiple testing correction with FDR < 0.05 as the threshold. After correction, 11 immune cell types remained significantly different between groups. Activated mast cells, which showed differences before correction, lost statistical significance. CD8 T cells exhibited the most prominent intergroup difference (FDR = 6.24 × 10^−8^). The FDR values of M1 and M2 macrophages were both 9.44 × 10^−7^, and the FDR of M0 macrophages was 5.06 × 10^−5^ (Figure 5d). Correlation analysis revealed that IFNG exhibited a remarkable positive correlation with CD8 T cells (r = 0.55, *p* < 0.05). (Figure 5e). In the end, analysis of differences in tumor scores demonstrated marked intergroup distinctions (*p* = 0.017) (Figure 5f).

### 3.7. Acquisition of 20 Immune Checkpoint Genes and Chemotherapy Drugs Beneficial to LRG

**Figure 6:**
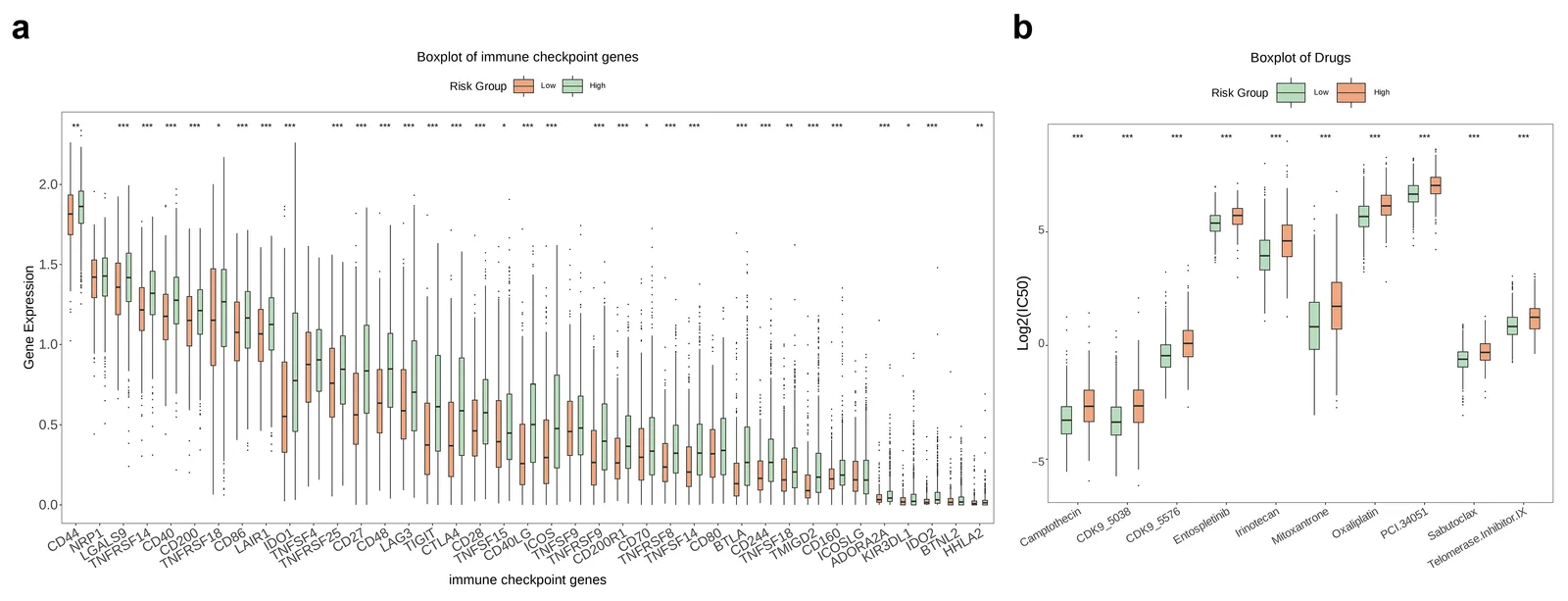
Analysis of immune checkpoint variants and drug sensitivity between high- and low-risk groups. (**a**) Immune checkpoint expression in high- and low-risk groups. Whiskers extend to the minimum and maximum values within 1.5 times the interquartile range, and scattered dots denote outliers. (**b**) Drug sensitivity analysis for high- and low-risk groups, * *p* < 0.05, ** *p* < 0.01, *** *p* < 0.001.

Using transcriptomic expression data, the Wilcoxon test (*p* < 0.05) showed that 33 immune checkpoint genes had statistically notable intergroup disparities in silico (Figure 6a).

Based on the BRCA samples from the TCGA-BRCA, the Wilcoxon test revealed that the IC_50_ values of 154 chemotherapeutic agents varied markedly in the two groups (*p* < 0.05). And camptothecin, entospletinib, irinotecan, among others, were listed as the top ten drugs. Specifically, these drugs exhibited elevated IC_50_ value in the HRG relative to the LRG (Figure 6b). Furthermore, the IC_50_ values were predicted using the GDSC database and the oncoPredict package, which only estimate drug sensitivity at the in vitro cell line level and do not equate to actual clinical therapeutic efficacy.

### 3.8. More PIC3KA Mutations Related to BRCA Somatic Cells and More CSMD3 and IRS Mutation Sites Related to HRG

**Figure 7:**
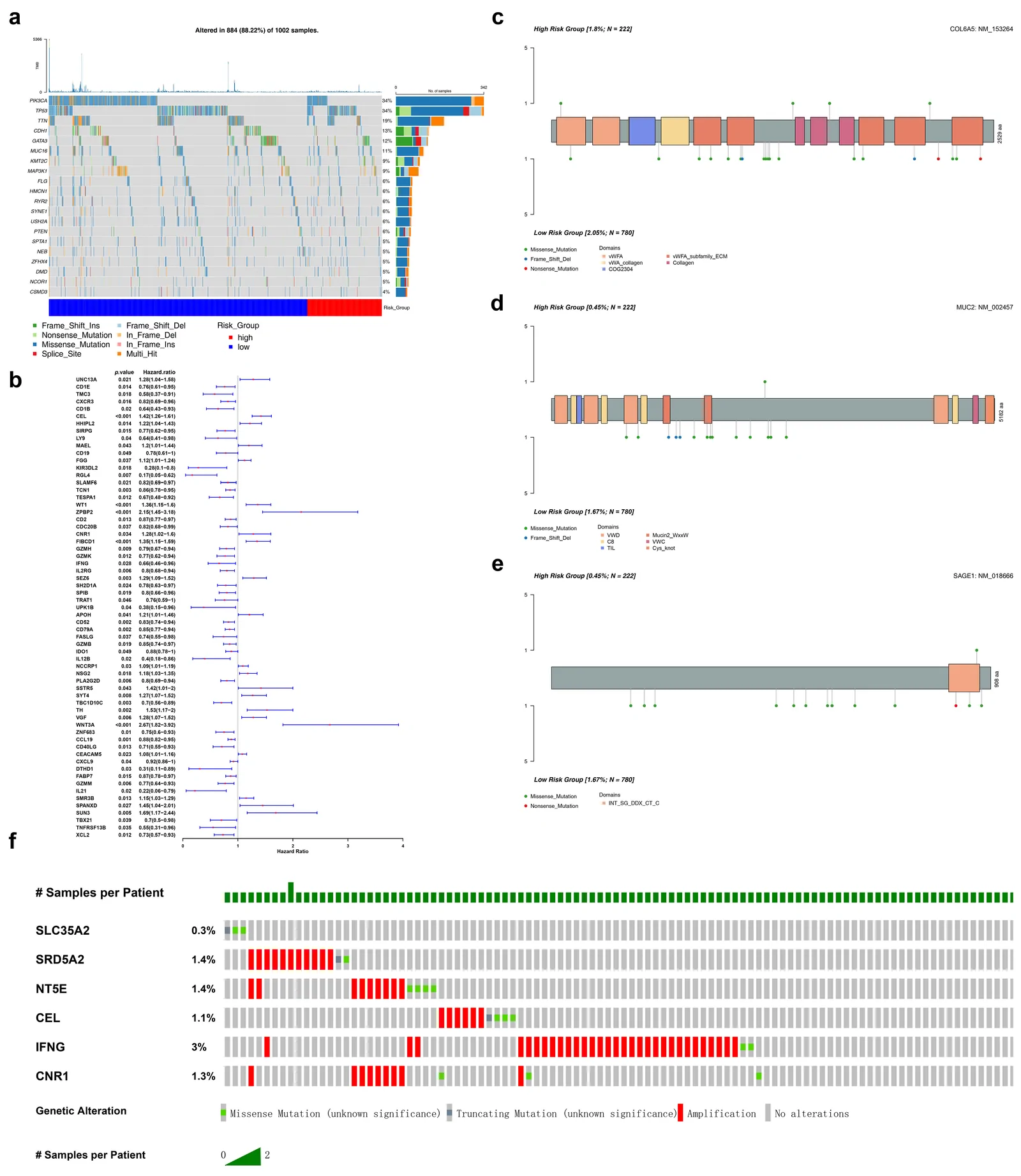
PIC3KA mutation analysis in TCGA-BRCA. (**a**) Cell mutation waterfall chart for high- and low-risk groups. (**b**) Key mutation gene single factor cox analysis forest plot. (**c**) COL6A5 mutation site lollipop plot. (**d**) MUC2 mutation site lollipop plot. (**e**) SAGE1 mutation site lollipop plot. (**f**) Prognosis gene mutation status. # stands for Number.

Somatic mutation analysis was performed on each BRCA sample from TCGA-BRCA. Among the top 20 frequently mutated genes, PIK3CA harbored the most frequent mutations (34%), predominantly missense mutations (Figure 7a). These mutation frequencies reflect database-annotated variants and represent computational summaries of sequencing data.

Differential analysis was performed on mutated genes between the two groups to identify differentially mutated genes. Through performing univariate Cox analysis, 63 KMGs were identified (Figure 7b). The HRG had significantly fewer mutation sites than the LRG on the top three mutated genes (COL6A5, MUC2, and SAGE1) (Figure 7c–e). Mutational analysis revealed that each prognostic gene had a small number of missense mutations, and IFNG had a higher proportion of amplification (Figure 7f).

### 3.9. Verification Identified Six Prognostic Genes with Significant Differences

**Figure 8:**
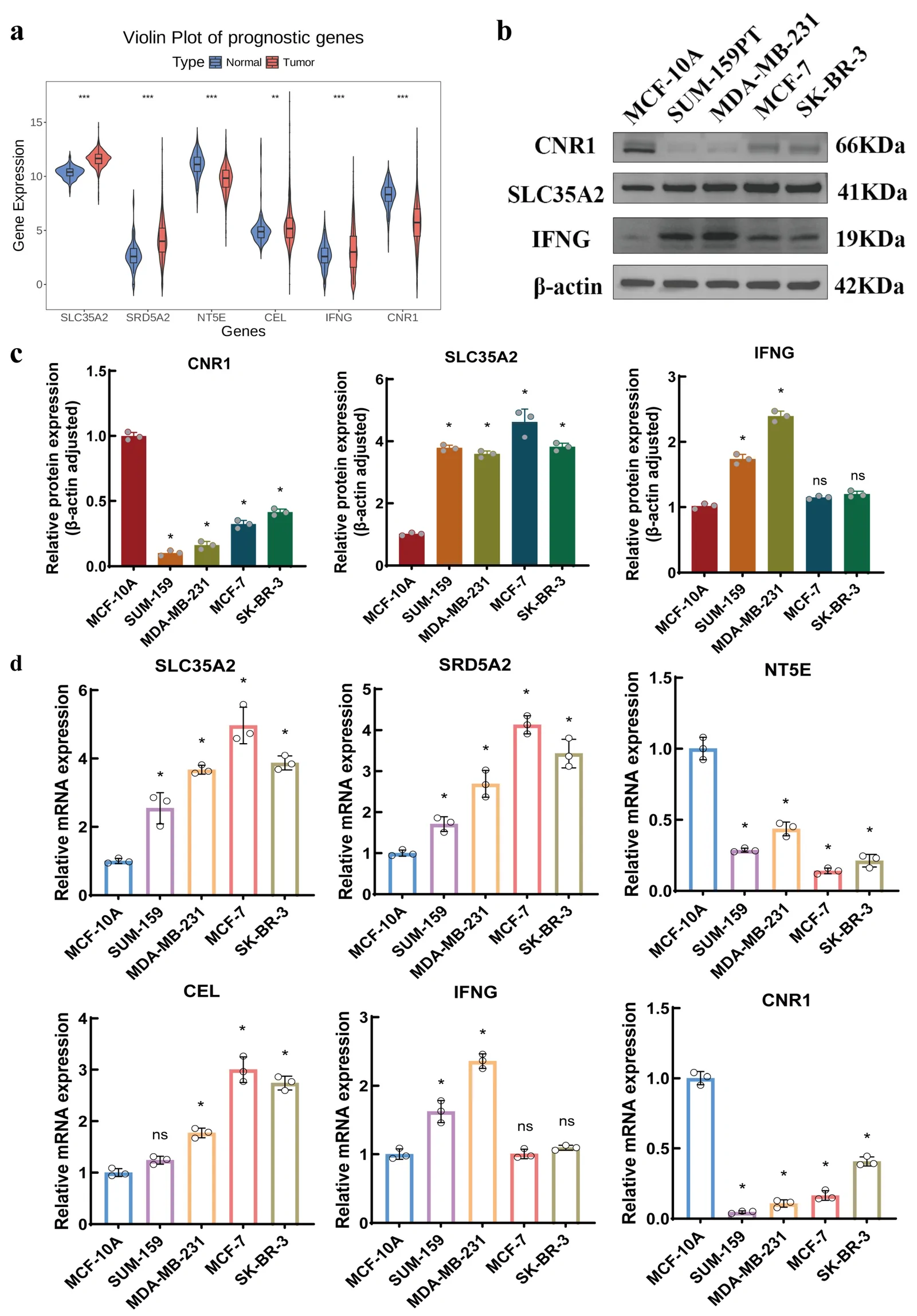
Expression validation of six asparagine metabolism-related prognostic genes. (**a**) Violin plots showing the expression distribution of prognostic genes (SLC35A2, SRD5A2, NT5E, CEL, IFNG, and CNR1) in breast tumor tissues (red) versus adjacent normal tissues (blue). *** *p* < 0.001, ** *p* < 0.01. (**b**) Representative Western blot bands showing the protein levels of CNR1, SLC35A2, and IFNG in human normal mammary epithelial MCF-10A cells and breast cancer cell lines, with β-actin as the loading control. (**c**) Bar graphs showing the quantitative gray-scale analysis of the blots in (**b**). Relative protein expression was normalized to β-actin. *p* < 0.05; ns, not significant. (**d**) mRNA expression profiles of six prognostic asparagine metabolism-related genes in breast cancer cell lines compared with MCF-10A. * Data are mean ± SD. * *p* < 0.05; ns, not significant.

To characterize the expression profiles of the six candidate prognostic genes in the TCGA-BRCA breast cancer cohort, an independent sample *t*-test was performed to compare their transcriptional abundances between breast carcinoma and matched normal mammary tissues, with a significance cutoff set at (*p* < 0.01). Statistical differences in expression were identified for all six genes across tumour and paracancerous specimens (Figure 8a). Specifically, SRD5A2, SLC35A2, CEL and IFNG were significantly upregulated in malignant breast tissues relative to normal controls, whereas NT5E and CNR1 were downregulated in tumour lesions.

We further detected the mRNA expression levels of the six prognostic genes in five breast cancer cell lines via real-time quantitative PCR (RT-qPCR) to verify their expression differences, thereby validating our previous bioinformatic findings in in vitro experiments (Figure 8d). Using the non-tumourigenic human mammary epithelial cell line MCF-10A as the baseline control, significantly elevated mRNA levels of SLC35A2, SRD5A2 and CEL were detected in all tested breast cancer cell lines (*p* < 0.05), following a descending expression rank: MCF-7 (Luminal subtype) > SK-BR-3 (HER2^+^ subtype) > MDA-MB-231 > SUM-159PT (triple-negative breast cancer, TNBC), indicative of subtype-preferred overexpression confined to the Luminal phenotype. By contrast, NT5E was markedly suppressed across all malignant cell lines compared with MCF-10A, with milder downregulation observed in TNBC cells versus Luminal and HER2-amplified counterparts. IFNG exhibited exclusive upregulation in two TNBC cell lines (SUM-159PT and MDA-MB-231, (*p* < 0.05), with comparable transcript abundance among MCF-7, SK-BR-3 and MCF-10A groups (ns, non-significant). CNR1 displayed the highest endogenous expression in normal MCF-10A cells and universal downregulation in all tumour cell lines; among malignant subtypes, SK-BR-3 retained relatively higher CNR1 expression while SUM-159PT presented the lowest level. Collectively, the six asparagine metabolism-related genes displayed distinct subtype-specific expression signatures across molecular subgroups of breast cancer, furnishing transcriptional evidence for subsequent in vivo histological verification and prognostic signature construction.

We selected SLC35A2, CNR1 and IFNG for further protein-level verification via Western blot, given their remarkably divergent and subtype-specific expression patterns across breast cancer cell subtypes. Quantitative grayscale analysis of Western blot results (Figure 8b,c) corroborated qRT-PCR observations at the translational level. Relative to MCF-10A, CNR1 protein was significantly depleted in all breast cancer cell lines (*p* < 0.05), with a descending protein abundance order: MCF-10A > SK-BR-3 > MCF-7 > MDA-MB-231 > SUM-159PT. SLC35A2 protein was consistently overexpressed in all molecular subtypes (*p* < 0.05), peaking in Luminal MCF-7 cells, followed by HER2^+^ SK-BR-3 and intermediate levels in TNBC cells. Consistent with mRNA trends, elevated IFNG protein was restricted exclusively to SUM-159PT and MDA-MB-231 (TNBC, (*p* < 0.05), with no significant intergroup differences between MCF-7, SK-BR-3 and MCF-10A (ns). The concordant expression patterns of the three genes at mRNA and protein levels jointly validated their subtype-biased expression features in distinct breast cancer subsets.

**Figure 9:**
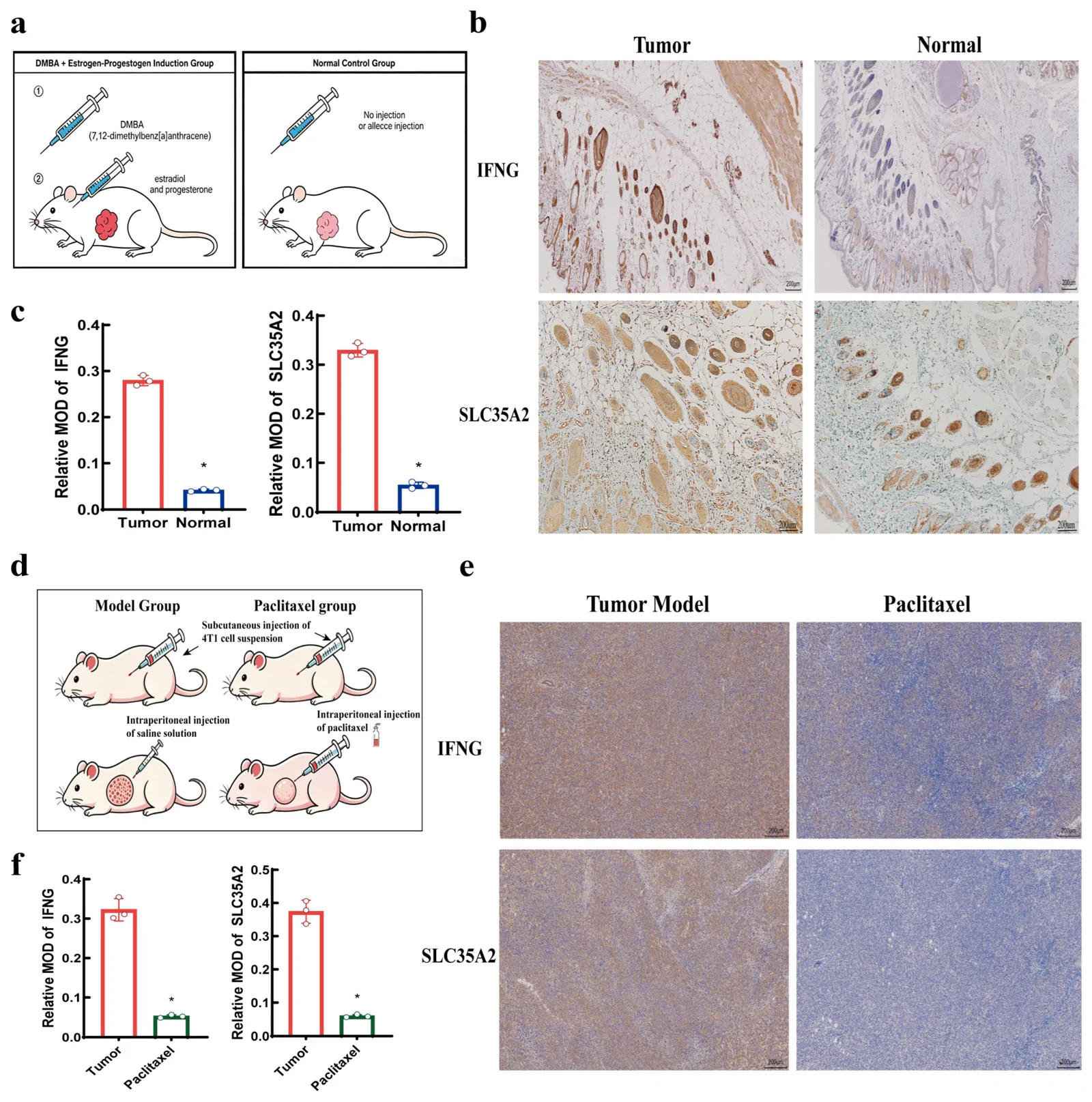
In vivo validation of IFNG and SLC35A2 expression in two breast cancer models. (**a**) Schematic illustration of the DMBA combined estrogen-progestogen-induced rat breast cancer model and the untreated normal control group. (**b**) Representative immunohistochemical (IHC) staining images of IFNG and SLC35A2 in DMBA-induced tumor tissues and adjacent normal tissues (scale bar: 200 μm). (**c**) Quantitative analysis of the relative mean optical density (MOD) of IFNG and SLC35A2 staining in tumor versus normal tissues (* *p* < 0.05 vs. normal group). (**d**) Schematic diagram of the 4T1 subcutaneous tumor-bearing mouse model, including the untreated model group and the paclitaxel-treated group. (**e**) Representative IHC staining images of IFNG and SLC35A2 in 4T1 tumor tissues from the untreated tumor model group and the paclitaxel-treated group (scale bar: 200 μm). (**f**) Quantitative analysis of the relative MOD of IFNG and SLC35A2 staining in tumor tissues of the 4T1 model versus the paclitaxel-treated group (* *p* < 0.05 vs. tumor model group).

We selected SLC35A2 and IFNG for verification of their differential protein expression at the tissue level via IHC staining in two in vivo models, since the two molecules represent key mediators involved in metabolic transport and immune regulatory pathways in our mechanistic hypothesis. Immunohistochemistry (IHC) staining of DMBA plus oestrogen/progesterone-induced spontaneous mammary carcinoma in SD rats recapitulated in vitro cellular findings (Figure 9a–c). Intense diffuse brown IHC positivity for SLC35A2 and IFNG was observed in malignant mammary lesions, whereas only faint sporadic staining was visible in paired normal mammary parenchyma, verifying that malignant transformation drives aberrant upregulation of both targets. In the 4T1 xenograft tumour model established in immunodeficient nude mice (Figure 9d–f), robust brown immunoreactivity of SLC35A2 and IFNG was similarly detected in untreated vehicle-group neoplasms, consistent with in vitro data: universal SLC35A2 overexpression in breast cancer and selective IFNG enrichment within TNBC. Notably, paclitaxel treatment markedly reduced both staining intensity and positive staining area of these two proteins within xenograft tumours. Mechanistically, it was speculated that SLC35A2 may have supported ASNS-catalyzed asparagine biosynthesis by transporting metabolic precursors to supplement substrate pools; meanwhile, IFNG may have transcriptionally regulated ASNS through the canonical JAK/STAT1 signaling cascade. These two molecules remodel tumour asparagine metabolism via discrete regulatory axes: substrate provision and inflammatory transcriptional modulation, respectively, and paclitaxel blocks dysregulated amino acid metabolism by suppressing their expression. Integrated evidence spanning in vitro cell phenotyping, chemically induced spontaneous rat mammary tumour tissues and pharmacologically intervened xenograft tumours confirms that SLC35A2 and IFNG serve as reliable biomarkers to discriminate normal from malignant mammary tissues and stratify breast cancer molecular subtypes, alongside promising utility for the pathological evaluation of chemotherapeutic responsiveness. These multilayered in vitro and in vivo experimental data lay solid empirical foundations for the construction of the asparagine metabolism-derived multigene prognostic signature in this study.

## 4. Discussion

Breast cancer is the most common malignant tumour in women, and heterogeneity among its molecular subtypes leads to marked interindividual differences in patient prognosis, immune phenotypes and chemotherapeutic sensitivity [25]. Metabolic reprogramming represents a core feature of tumour malignant transformation. Aberrant asparagine metabolism persistently promotes breast cancer proliferation, invasion and metastasis and has become a research hotspot in tumour metabolism. Most existing studies focus on ASNS, a single key enzyme in this pathway, and merely confirm that its overexpression correlates with poor prognosis. Few efforts have constructed multi-gene prognostic models from the perspective of the entire metabolic pathway, which limits our understanding of the comprehensive regulatory effects of asparagine metabolism on the tumour microenvironment and drug sensitivity [26,27]. Accordingly, we do not merely focus on individual metabolic enzymes, and propose that the asparagine-related gene network jointly regulates substrate supply, tumour immune crosstalk and chemosensitivity. Owing to breast cancer molecular heterogeneity and limitations of single-enzyme studies, we screened candidate genes from GeneCards. Using TCGA-BRCA and independent GEO transcriptomic datasets, we built a risk scoring model to achieve improved prognostic stratification for breast cancer patients.

Using sequential screening consisting of univariate Cox regression, LASSO penalized regression and multivariate Cox regression, we identified six core prognostic genes from 334 candidate asparagine metabolism-related genes: SLC35A2, SRD5A2, NT5E, CEL, IFNG and CNR1. The risk score model built upon these six genes achieved effective risk stratification in the TCGA training cohort and the GSE96058 validation cohort, with AUC values exceeding 0.70 for 1-year, 3-year and 5-year survival prediction. SRD5A2 and IFNG were upregulated in patients of the low-risk group, whereas SLC35A2, NT5E, CEL and CNR1 were downregulated. Additionally, the risk score was significantly correlated with clinical stage, T stage and N stage.

Collectively, these findings confirm the reliable discriminatory ability and independent prognostic value of our asparagine metabolism-associated signature. A multi-gene strategy is biologically justified: asparagine metabolism is not regulated merely by the single rate-limiting enzyme ASNS, but coordinated by a distributed regulatory network integrating substrate supply, energy coupling, immune regulation and redox homeostasis to support tumour anabolic metabolism [12]. Unlike previous studies focusing solely on ASNS-mediated enzymatic reactions, the six hub genes identified here engage in diverse processes such as substrate transport, glycolipid metabolism, steroid signalling and immune regulation. These genes interact to form an integrated network reflecting overall metabolic reprogramming and breast cancer progression. Our results offer new insights into molecular subtyping, individualized prognostic stratification and targeted therapeutic development for breast cancer.

Functional enrichment clustering segregated the six hub genes into three interconnected modules: metabolic reprogramming, immune-metabolic crosstalk and redox homeostasis regulation, which synergistically orchestrate asparagine metabolic rewiring and breast cancer progression. Importantly, the functions of these modules are not isolated; instead, they form positive regulatory loops. Metabolic flux shapes the characteristics of the tumour immune microenvironment, and the immune microenvironment in turn remodels cellular redox status and tumour drug sensitivity. The metabolic reprogramming module comprises SLC35A2, CEL and SRD5A2, which furnish sufficient substrates and energy pools for asparagine biosynthesis by remodelling intracellular anabolic metabolism. As an essential nucleoside-sugar transporter, aberrant glycosylation mediated by SLC35A2 triggers ERK cascade activation and subsequent epithelial-mesenchymal transition [28]. Existing literature has demonstrated that post-translational glycosylation stabilizes ASNS protein and potentiates its catalytic activity, enabling persistent asparagine synthesis under nutrient deprivation [29], which provides mechanistic underpinnings for SLC35A2-mediated asparagine metabolism in our research. CEL fuels tumour anabolism by promoting lipolysis; acetyl-CoA generated from lipid breakdown acts as a vital precursor for amino acid synthesis and improves metabolic fitness of cancer cells under nutritional stress [30,31]. SRD5A2 modulates steroid signalling to upregulate multiple amino acid transporters, thereby enhancing cellular amino acid uptake and intracellular asparagine storage indirectly [32].

The immune-metabolic crosstalk module consists of IFNG and NT5E, serving as an indispensable molecular bridge linking tumour immune microenvironment and asparagine metabolic reprogramming. This module receives upstream metabolic signals, such as asparagine abundance sensed via mTORC1, and translates these signals into immune effector programs, thereby completing the signalling circuit of the asparagine metabolism multi-dimensional regulatory axis (AMMA axis) [10]. IFN-γ is predominantly secreted by activated NK cells and cytotoxic T lymphocytes, which drives macrophage polarization towards antitumour M1 phenotype and upregulates tumoral IDO expression. Tryptophan depletion induced by elevated IDO reciprocally stimulates ASNS transcription to boost asparagine synthesis, empowering cancer cells to overcome immune pressure and sustain malignant proliferation [33,34]. By contrast, NT5E catalyses adenosine production to suppress cytotoxic function of CD8^+^ T cells; meanwhile, NT5E triggers mTORC1 signalling to transcriptionally activate ASNS, further facilitating metabolic reprogramming and tumour immune escape [8,35]. Our results demonstrated robust IFNG expression accompanied by abundant intratumoral CD8^+^ T cell infiltration in low-risk patients, indicative of potent antitumour immune surveillance capable of restraining asparagine-dependent tumour proliferation.

CNR1 is the core regulator of redox balance and ferroptosis, which mediates adaptive tumour metabolism by remodelling membrane lipid homeostasis. CNR1 acts as a terminal node on the asparagine metabolism multi-dimensional regulatory axis (AMMA axis). It integrates dual input signals derived from metabolism and immunity to determine cell fate under nutrient stress, thus forming a complete regulatory circuit linking metabolic demand to adaptive responses. CNR1 modulates fatty acid desaturase expression to reshape lipid composition of plasma membrane and dictate cellular susceptibility to ferroptosis [36]. When deprived of glutamine within the microenvironment, tumour cells resort to asparagine as an alternative nitrogen source to maintain proliferation and metabolic stability [37]. CNR1-dependent lipid remodelling confers ferroptosis resistance and enhanced antioxidative capacity, strengthening metabolic plasticity of malignant cells [38]. Significantly downregulated CNR1 in low-risk subgroups implies inferior metabolic flexibility, restricting compensatory asparagine-fuelled tumour growth under nutrient-limited conditions.

Subtype heterogeneity fundamentally underpins divergent metabolic phenotypes across breast cancer subgroups. We propose that SLC35A2 supplies glycosylation precursors to stabilize ASNS protein via post-translational modification. IFNG upregulates ASNS transcription through the JAK/STAT1 pathway, and CNR1 indirectly modulates ASNS by regulating ATF4 signalling activity. SRD5A2, CEL and NT5E cooperatively balance carbon-nitrogen homeostasis to sustain the intracellular asparagine pool. This mechanistic model explains why our multi-gene signature outperforms the single biomarker ASNS [11]. Consistent with our in vitro and in vivo experimental validation, the six hub genes exhibit distinct subtype-restricted expression patterns, resulting in variable asparagine dependency among different breast cancer subtypes. Luminal tumours feature high expression of SLC35A2, SRD5A2 and CEL, which facilitate asparagine metabolism via activated glycolipid and steroid signalling pathways; TNBC tumours selectively overexpress IFNG to drive metabolic rewiring through inflammatory-metabolic coupling. Ubiquitous downregulation of CNR1 and NT5E across all subtypes relieves intrinsic metabolic suppression to accelerate tumour advancement. Stratification analysis coupled with molecular subtyping revealed that high-risk patients were predominantly enriched within the TNBC subtype, characterized by intensified inflammation-triggered asparagine biosynthesis, advanced malignancy and unfavourable prognosis, whereas low-risk cases were dominated by luminal tumours with lower metabolic addiction and superior clinical outcomes. Collectively, this metabolism-based prognostic signature simultaneously enables molecular subtyping and risk stratification by faithfully recapitulating subtype-specific metabolic landscapes and prognostic disparities.

It should be noted that our inferences concerning the ASNS/JAK/STAT1 axis and paclitaxel-regulated asparagine metabolism are based only on bioinformatic predictions and expression validation in cells and tissues. Without gene manipulation and metabolomic evidence, these mechanistic results need to be interpreted with caution. From the perspective of metabolic regulatory mechanisms, none of the screened genes exclusively function as core enzymes for asparagine synthesis; instead, they assemble a multi-layered and interconnected regulatory network. SLC35A2 is speculated to participate in transporting precursor substrates required for asparagine biosynthesis. IFNG may transcriptionally upregulate ASNS by targeting its promoter through the JAK/STAT1 signaling cascade, thus potentially promoting asparagine production CNR1, which indirectly modulates ASNS expression through altering the activity of the ATF4 pathway. SRD5A2, CEL and NT5E synergistically remodel asparagine metabolism in an auxiliary manner by balancing intracellular carbon and nitrogen homeostasis. Collectively, these mechanistic characteristics account for the superior predictive performance and biological robustness of our six-gene signature compared with previously reported single-gene or single-pathway metabolic biomarkers for breast cancer prognosis [25]. Benefiting from its multi-dimensional regulatory features, this signature comprehensively reflects heterogeneous asparagine dependence across distinct breast cancer molecular subtypes and overcomes the poor universality and limited applicability of conventional single-metabolic biomarkers.

Tumour-infiltrating immune cells are critical modulators of cancer metabolism and therapeutic responsiveness. The asparagine metabolism multi-dimensional regulatory axis (AMMA axis) reveals links between tumour immune microenvironment and drug sensitivity. Tumour and immune cells compete for asparagine. Tumours with activated AMMA axis prevail in competition, acquire metabolic plasticity and develop chemoresistance [39]. Distinct immune infiltration landscapes were observed between high- and low-risk cohorts: high-risk tumours were enriched with protumoural M0 macrophages that establish an immunosuppressive microenvironment, whereas low-risk lesions accumulated activated memory CD4^+^ T cells associated with robust antitumour immunity [40,41]. Immune cells compete for nutritive asparagine in the interstitial microenvironment to constrain tumour metabolism and proliferation. Drug susceptibility analysis further indicated superior sensitivity to irinotecan, oxaliplatin, sabutoclax and multiple other chemo- and targeted agents among low-risk patients, attributed to inherently weak asparagine metabolism and limited metabolic adaptability, rendering these tumours susceptible to drug-initiated metabolic stress and apoptotic cell death [42]. These bioinformatic findings provide rational evidence for individualized chemotherapy guided by our risk stratification system.

Several limitations should be acknowledged in the present work. Although the six prognostic genes were screened from asparagine metabolism-related candidates, direct metabolomic data are lacking to confirm that the risk stratification faithfully mirrors variations in tumour asparagine metabolism, which represents a priority for future investigation. Our validation is confined to gene expression profiling. Further functional assays, including CRISPR-mediated gene manipulation, ASNS rescue experiments and metabolomic profiling, are required to establish causal relationships between these hub genes and asparagine metabolism and dissect the precise molecular mechanisms underlying ASNS regulation by SLC35A2, IFNG and other targets. The prognostic model was developed using retrospective TCGA and GEO datasets. Multicentre prospective cohort validation is still needed to consolidate its clinical utility. Additionally, estimates of immune infiltration, drug susceptibility and mutational landscapes rely on computational predictions from public databases. Potential biases embedded in relevant algorithms may interfere with result interpretation, so experimental verification is necessary before clinical translation. Future efforts will adopt LC-MS/MS to quantify asparagine and related metabolites in patient tissues and isogenic cell models. CRISPR-Cas9 editing will be applied to assess alterations in asparagine metabolic flux. Prospective multicentre cohorts will also be recruited to validate the model and facilitate the translational application of these metabolic biomarkers.

## 5. Conclusions

In this study, six prognostic genes (SLC35A2, SRD5A2, NT5E, CEL, IFNG, CNR1) were identified from GeneCards-derived asparagine metabolism-related candidates via transcriptomic mining and multi-dimensional bioinformatic analyses to construct a multi-gene transcriptional risk signature. This model achieves reliable prognostic stratification of breast cancer patients; its risk score correlates well with molecular subtypes, showing robust discriminatory capability and promising translational potential. These six genes exhibit subtype-specific expression: SLC35A2, SRD5A2 and CEL are upregulated in luminal breast cancer, IFNG is enriched in triple-negative tumours, while CNR1 and NT5E are consistently downregulated across all subtypes. Animal experiments confirmed paclitaxel suppresses tumoural SLC35A2 and IFNG expression, supporting their utility as biomarkers for subtyping and chemotherapeutic response evaluation. Network analysis indicated these genes interact with asparagine metabolic hubs through substrate transport, inflammatory regulation and compensatory metabolism, and the risk score correlates with microenvironment remodelling and drug sensitivity. Collectively, this work clarifies expression heterogeneity of asparagine-related genes and offers a stratification tool for breast cancer, guiding future therapies targeting asparagine metabolic networks.

## Figures and Tables

**Table 1:** Primer sequences used for RT-qPCR.

Genes	Forward Primer	Reverse Primer	Amplicon Length (bp)	NCBI GenBank Accession
SLC35A2	GAGGTGCAGCCAAAGCCATA	GAAAGGGCTCCGTGATGAGG	132	NM_005660.3
SRD5A2	GGGGCTGTGTGCTGGAAATA	GCACCAGGAGTGGCATCTAA	123	NM_000348.4
NT5E	TGGTGGAGATGGGTTCCAGA	CCGACCTTCAACTGCTGGAT	127	NM_002526.4
CEL	AAGAGGCCACAAGAGTGGGA	AGGGGTATGAGGCTTTATTCAGG	75	NM_001807.6
IFNG	TGAATGTCCAACGCAAAGCA	ACTGGGATGCTCTTCGACCT	123	NM_000619.3
CNR1	GTGTTCCACCGCAAAGATAGC	GGGGCCTGTGAATGGATATGT	130	NM_001160259.3

## Data Availability

The TCGA-BRCA dataset is available from The Cancer Genome Atlas via the GDC Data Portal (https://portal.gdc.cancer.gov/ accessed on 21 April 2025) and the UCSC Xena Browser (https://xenabrowser.net/datapages/ accessed on 21 April 2025). The GSE96058 dataset is available from the Gene Expression Omnibus database (https://www.ncbi.nlm.nih.gov/geo/query/acc.cgi?acc=GSE96058 accessed on 21 April 2025). All WB and IHC raw datasets generated in this study are available in the Supplementary File and figshare: raw datasets. figshare. Dataset. https://doi.org/10.6084/m9.figshare.32800803 accessed on 26 June 2026.

## Supplementary Materials

The following supporting information can be downloaded at:  https://www.mdpi.com/article/10.3390/biology15161441/s1, Table S1: Reference gene collections used for GSEA. Table S2: PH assumption test results for candidate prognostic genes. Table S3: PH assumption test results for the six prognostic genes. Table S4: coefficients of the six-gene prognostic model. Table S5: GSEA enrichment results. Supplementary Raw WB Image File.

## List of Abbreviations

Abbreviation	Full term
AMRGs	asparagine metabolism-related genes
ANOVA	analysis of variance
ASNS	asparagine synthetase
ATCC	American Type Culture Collection
ATF4	activating transcription factor 4
AUC	area under the curve
BLAST	Basic Local Alignment Search Tool
BP	biological process
BRCA	breast cancer
CBio/cBioPortal	cBio Cancer Genomics Portal
CC	cellular component
cDNA	complementary DNA
CEL	carboxyl ester lipase
CGs	candidate genes
CI	confidence interval
CIBERSORT	Cell-type Identification By Estimating Relative Subsets Of RNA Transcripts
CNR1	cannabinoid receptor 1
CoA	coenzyme A
CPGs	candidate prognostic genes
CSMD3	CUB and sushi multiple domains 3
DAB	3,3′-diaminobenzidine
DEGs	differentially expressed genes
DMBA	7,12-dimethylbenz[a]anthracene
DMEM	Dulbecco’s modified Eagle medium
DMGs	differentially mutated genes
DNA	deoxyribonucleic acid
ECL	enhanced chemiluminescence
EMT	epithelial-mesenchymal transition
ER	estrogen receptor
ERK	extracellular signal-regulated kinase
ESTIMATE	Estimation of Stromal and Immune cells in Malignant Tumor tissues using Expression data
FBS	fetal bovine serum
FDR	false discovery rate
FLG2	filaggrin 2
GDSC	Genomics of Drug Sensitivity in Cancer
GEO	Gene Expression Omnibus
GLS	glutaminase
GLUL	glutamate-ammonia ligase
GO	Gene Ontology
GSEA	gene set enrichment analysis
HER2	human epidermal growth factor receptor 2
HR	hazard ratio
HRG	high-risk group
HRP	horseradish peroxidase
IACUC	Institutional Animal Care and Use Committee
IC50	half-maximal inhibitory concentration
IDO	indoleamine 2,3-dioxygenase
IFN-γ	interferon-gamma
IFNG	interferon gamma
IHC	immunohistochemistry
IPFs	independent prognostic factors
IRS4	insulin receptor substrate 4
JAK/STAT1	Janus kinase/signal transducer and activator of transcription 1
KEGG	Kyoto Encyclopedia of Genes and Genomes
K-M	Kaplan–Meier
KMGs	key mutated genes
LASSO	least absolute shrinkage and selection operator
LRG	low-risk group
MF	molecular function
MOD	mean optical density
mTORC1	mechanistic target of rapamycin complex 1
NCBI	National left for Biotechnology Information
NES	normalized enrichment score
NK	natural killer
ns	not significant
NT5E	5′-nucleotidase ecto
OD260/OD280	optical density ratio at 260 and 280 nm
PBS	phosphate-buffered saline
PCR	polymerase chain reaction
PH	proportional hazards
PHGDH	phosphoglycerate dehydrogenase
PIK3CA	phosphatidylinositol-4,5-bisphosphate 3-kinase catalytic subunit alpha
PPI	protein-protein interaction
PR	progesterone receptor
PSPH	phosphoserine phosphatase
PVDF	polyvinylidene fluoride
RIPA	radioimmunoprecipitation assay
RNA	ribonucleic acid
ROC	receiver operating characteristic
RT-qPCR/qRT-PCR	reverse transcription quantitative polymerase chain reaction
SD	standard deviation; Sprague-Dawley in SD rats
SDICs	significantly differential immune cells
SDS-PAGE	sodium dodecyl sulfate-polyacrylamide gel electrophoresis
SLC35A2	solute carrier family 35 member A2
SLC38A3	solute carrier family 38 member 3
SPF	specific pathogen-free
SRD5A2	steroid 5 alpha-reductase 2
TCGA	The Cancer Genome Atlas
TCGA-BRCA	The Cancer Genome Atlas breast cancer cohort
TNBC	triple-negative breast cancer
UCSC	University of California, Santa Cruz

## References

[1] Sung H., Ferlay J., Siegel R.L., Laversanne M., Soerjomataram I., Jemal A., Bray F. (2021). Global Cancer Statistics 2020: GLOBOCAN Estimates of Incidence and Mortality Worldwide for 36 Cancers in 185 Countries. CA Cancer J. Clin..

[2] Siegel R.L., Miller K.D., Fuchs H.E., Jemal A. (2022). Cancer statistics, 2022. CA Cancer J. Clin..

[3] Zhai Z., Zheng Y., Yao J., Liu Y., Ruan J., Deng Y., Zhou L., Zhao P., Yang S., Hu J. (2020). Evaluation of Adjuvant Treatments for T1 N0 M0 Triple-Negative Breast Cancer. JAMA Netw. Open.

[4] Siegel R.L., Kratzer T.B., Giaquinto A.N., Sung H., Jemal A. (2025). Cancer statistics, 2025. CA Cancer J. Clin..

[5] Britt K.L., Cuzick J., Phillips K.A. (2020). Key steps for effective breast cancer prevention. Nat. Rev. Cancer.

[6] Liang J., Wang X., Yang J., Sun P., Sun J., Cheng S., Liu J., Ren Z., Ren M. (2023). Identification of disulfidptosis-related subtypes, characterization of tumor microenvironment infiltration, and development of a prognosis model in breast cancer. Front. Immunol..

[7] Chen W., Qin Y., Qiao L., Liu X., Gao C., Li T.-R., Luo Y., Li D., Yan H., Han L. (2025). FAM50A drives breast cancer brain metastasis through interaction with C9ORF78 to enhance L-asparagine production. Sci. Adv..

[8] Yuan Q., Yin L., He J., Zeng Q., Liang Y., Shen Y., Zu X. (2024). Metabolism of asparagine in the physiological state and cancer. Cell Commun. Signal..

[9] Shen X., Jain A., Aladelokun O., Yan H., Gilbride A., Ferrucci L.M., Lu L., Khan S.A., Johnson C.H. (2022). Asparagine, colorectal cancer, and the role of sex, genes, microbes, and diet: A narrative review. Front. Mol. Biosci..

[10] Xu Y., Shi T., Cui X., Yan L., Wang Q., Xu X., Zhao Q., Xu X., Tang Q., Tang H. (2021). Asparagine reinforces mTORC1 signaling to boost thermogenesis and glycolysis in adipose tissues. EMBO J..

[11] Li C., Mao Y., Hu J., Su C., Li M., Tan H. (2024). Integrating machine learning and multi-omics analysis to develop an asparagine metabolism immunity index for improving clinical outcome and drug sensitivity in lung adenocarcinoma. Immunol. Res..

[12] Jiang J., Batra S., Zhang J. (2021). Asparagine: A Metabolite to Be Targeted in Cancers. Metabolites.

[13] Tan Z., Boyapati K., Tressler C.M., Jenkinson N.M., Glunde K. (2024). Glutamine transporter SLC38A3 promotes breast cancer metastasis via Gsk3β/β-catenin/EMT pathway. Cancer Lett..

[14] Yuan Q., Wang Q., Li J., Yin L., Liu S., Zu X., Shen Y. (2025). CCT196969 inhibits TNBC by targeting the HDAC5/RXRA/ASNS axis to down-regulate asparagine synthesis. J. Exp. Clin. Cancer Res..

[15] Zeng X., Shi G., He Q., Zhu P. (2021). Screening and predicted value of potential biomarkers for breast cancer using bioinformatics analysis. Sci. Rep..

[16] Liu S., Wang Z., Zhu R., Wang F., Cheng Y., Liu Y. (2021). Three Differential Expression Analysis Methods for RNA Sequencing: Limma, EdgeR, DESeq2. J. Vis. Exp..

[17] Zhou W., Li H., Zhang J., Liu C., Liu D., Chen X., Ouyang J., Zeng T., Peng S., Ouyang F. (2025). Identification and mechanism analysis of biomarkers related to butyrate metabolism in COVID-19 patients. Ann. Med..

[18] Yang L., Qu Q., Hao Z., Sha K., Li Z., Li S. (2022). Powerful Identification of Large Quantitative Trait Loci Using Genome-Wide R/glmnet-Based Regression. J. Hered..

[19] Zheng Y., Wen Y., Cao H., Gu Y., Yan L., Wang Y., Wang L., Zhang L., Shao F. (2021). Global Characterization of Immune Infiltration in Clear Cell Renal Cell Carcinoma. OncoTargets Ther..

[20] Xue Q.Q., Liu C.H., Li Y. (2024). Decoding the anti-hypertensive mechanism of alpha-mangostin based on network pharmacology, molecular docking and experimental validation. Mol. Med..

[21] Hu X., Ni S., Zhao K., Qian J., Duan Y. (2022). Bioinformatics-Led Discovery of Osteoarthritis Biomarkers and Inflammatory Infiltrates. Front. Immunol..

[22] Lyu L., Jia H., Liu Q. (2024). Individualized lipid profile in urine-derived extracellular vesicles from clinical patients with Mycobacterium tuberculosis infections. Front. Microbiol..

[23] Maeser D., Gruener R.F., Huang R.S. (2021). oncoPredict: An R package for predicting in vivo or cancer patient drug response and biomarkers from cell line screening data. Brief. Bioinform..

[24] Shen A., Ye Y., Chen F., Xu Y., Zhang Z., Zhao Q., Zeng Z.-L. (2022). Integrated multi-omics analysis identifies CD73 as a prognostic biomarker and immunotherapy response predictor in head and neck squamous cell carcinoma. Front. Immunol..

[25] Zhang X. (2023). Molecular Classification of Breast Cancer: Relevance and Challenges. Arch. Pathol. Lab. Med..

[26] Qin C., Yang X., Zhan Z. (2020). High Expression of Asparagine Synthetase Is Associated with Poor Prognosis of Breast Cancer in Chinese Population. Cancer Biother. Radiopharm..

[27] Nishikawa G., Kawada K., Hanada K., Maekawa H., Itatani Y., Miyoshi H., Taketo M.M., Obama K. (2022). Targeting Asparagine Synthetase in Tumorgenicity Using Patient-Derived Tumor-Initiating Cells. Cells.

[28] Yang X., Tao Y., Xu Y., Cai W., Shao Q. (2024). SLC35A2 expression drives breast cancer progression via ERK pathway activation. Febs J..

[29] Zhang J., Fan J., Venneti S., Cross J.R., Takagi T., Bhinder B., Djaballah H., Kanai M., Cheng E.H., Judkins A.R. (2014). Asparagine plays a critical role in regulating cellular adaptation to glutamine depletion. Mol. Cell.

[30] Elia I., Rossi M., Stegen S., Broekaert D., Doglioni G., van Gorsel M., Fendt S.M. (2019). Breast cancer cells rely on environmental pyruvate to shape the metastatic niche. Nature.

[31] Brekke R.S., Gravdal A., El Jellas K., Curry G.E., Lin J., Wilhelm S.J., Molven A. (2024). Common single-base insertions in the VNTR of the carboxyl ester lipase (CEL) gene are benign and also likely to arise somatically in the exocrine pancreas. Hum. Mol. Genet..

[32] Dai C., Heemers H., Sharifi N. (2017). Androgen Signaling in Prostate Cancer. Cold Spring Harb. Perspect. Med..

[33] Munn D.H., Mellor A.L. (2016). IDO in the Tumor Microenvironment: Inflammation, Counter-Regulation, and Tolerance. Trends Immunol..

[34] Sun L., Kees T., Almeida A.S., Liu B., He X.-Y., Ng D., Han X., Spector D.L., McNeish I.A., Gimotty P. (2021). Activating a collaborative innate-adaptive immune response to control metastasis. Cancer Cell.

[35] Chen L., Qi T., Zhang B., Wang X., Zheng M. (2025). NT5E (CD73) as a prognostic biomarker and therapeutic target associated with immune infiltration in lung adenocarcinoma. Sci. Rep..

[36] Li P., Lin Q., Sun S., Yang N., Xia Y., Cao S., Zhang W., Li Q., Guo H., Zhu M. (2022). Inhibition of cannabinoid receptor type 1 sensitizes triple-negative breast cancer cells to ferroptosis via regulating fatty acid metabolism. Cell Death Dis..

[37] Knott S.R.V., Wagenblast E., Khan S., Kim S.Y., Soto M., Wagner M., Turgeon M.-O., Fish L., Erard N., Gable A.L. (2018). Asparagine bioavailability governs metastasis in a model of breast cancer. Nature.

[38] Stockwell B.R., Jiang X., Gu W. (2020). Emerging Mechanisms and Disease Relevance of Ferroptosis. Trends Cell Biol..

[39] Wu J., Li G., Li L., Li D., Dong Z., Jiang P. (2021). Asparagine enhances LCK signalling to potentiate CD8+ T-cell activation and anti-tumour responses. Nat. Cell Biol..

[40] Liu D., Vadgama J., Wu Y. (2021). Basal-like breast cancer with low TGFbeta and high TNFalpha pathway activity is rich in activated memory CD4 T cells and has a good prognosis. Int. J. Biol. Sci..

[41] Xu X., Wang J. (2023). Prognostic prediction and multidimensional dissections of a macrophages M0-related gene signature in liver cancer. Front. Endocrinol..

[42] Hu Y., Yagüe E., Zhao J., Wang L., Bai J., Yang Q., Pan T., Zhao H., Liu J., Zhang J. (2018). Sabutoclax, pan-active BCL-2 protein family antagonist, overcomes drug resistance and eliminates cancer stem cells in breast cancer. Cancer Lett..

