# Supplementary material for: Bioinformatic Identification and Experimental Validation of a Prognostic Transcriptional Signature Derived from Asparagine Metabolism-Related Genes in Breast Cancer
Source: Biology (Basel). 2026 Aug 21;15(16):1441. doi: 10.3390/biology15161441 (PMC13509382; doi:10.3390/biology15161441)

Supplementary Explanation for Unedited Raw Western Blot Images

Manuscript ID: biology-4421333

Figure involved: Figure 8b

1. Marker information

Prestained protein molecular weight marker was used to indicate protein size.

2. Lane layout (left to right)

Lane 1: MCF-10A

Lane 2: SUM-159PT

Lane 3: MDA-MB-231

Lane 4: MCF-7

Lane 5: SK-BR-3

3. Target protein and internal reference

Target protein: SLC35A2 / IFNG / CNR1

Internal reference: β-actin

Molecular weight of target protein: IFNG : 22kDa; SLC35A2: 41kDa; CNR1: 66kDa; β-actin: 42 kDa.

4. Separate membrane statement

The molecular weights of target proteins and β-actin are very close. To prevent band overlap and ensure accurate gray value quantification, all samples were loaded, electrophoresed and transferred under identical experimental conditions. The proteins were transferred onto two independent PVDF membranes, followed by separate primary antibody incubation, secondary antibody incubation and chemiluminescence exposure.

5. Information about biological replicates

All quantitative data in the manuscript are derived from three independent biological replicates (n=3). For each replicate, separate blots for target and reference protein are provided. All uploaded TIFF files are complete, uncropped original images exported directly from the chemiluminescence imaging system. No brightness, contrast or digital modification was applied to any raw blot.

6. Image processing note

Only the cropped composite figures displayed in the manuscript are used for layout presentation. All full intact raw unedited blots are submitted for technical verification.

Fig.8B

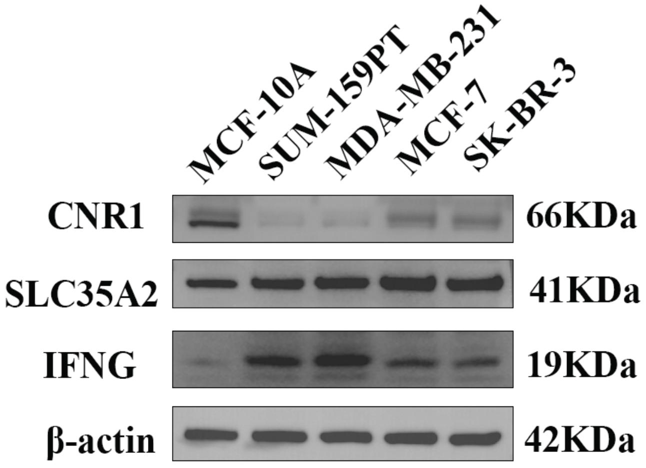

Sourse Data Fig.8B： β -actin: 42 kDa

Repetition 1

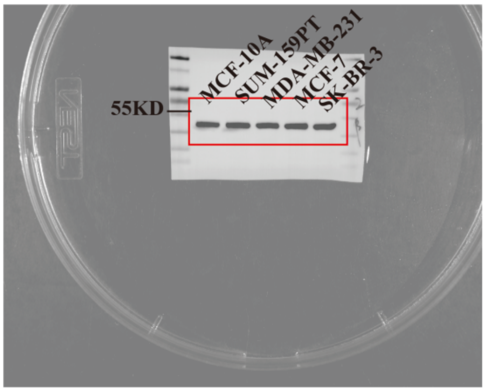

Repetition 2

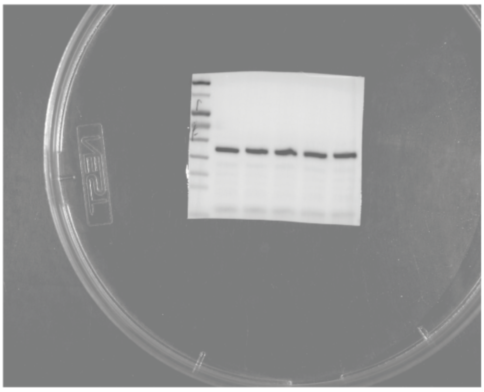

Repetition 3

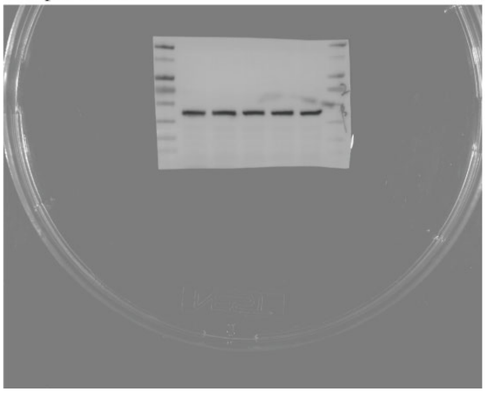

Sourse Data Fig.8B： SLC35A2: 41kDa

Repetition 1

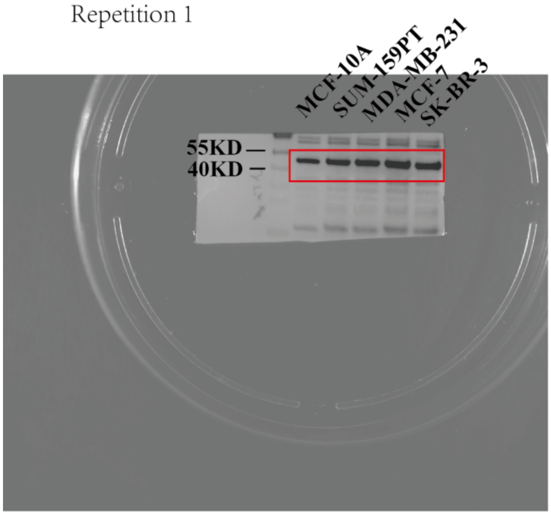

Repetition 2

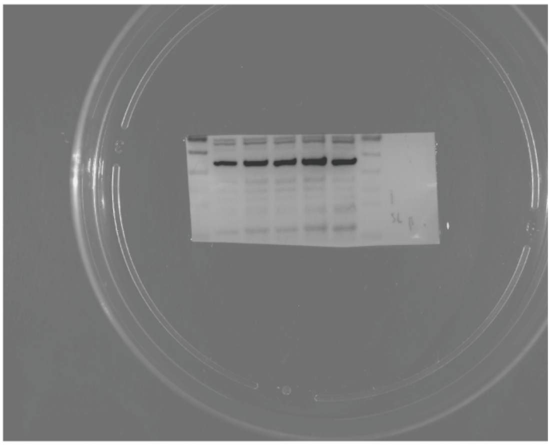

Repetition 3

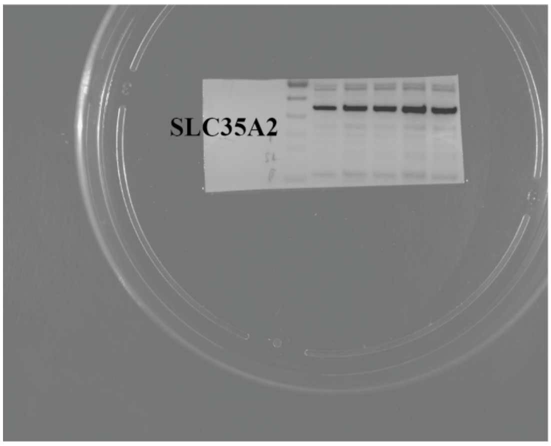

Sourse Data Fig.8B： IFNG : 19-22kDa

Repetition 1

Due to unclear markers after merging, white light images were added as supplements

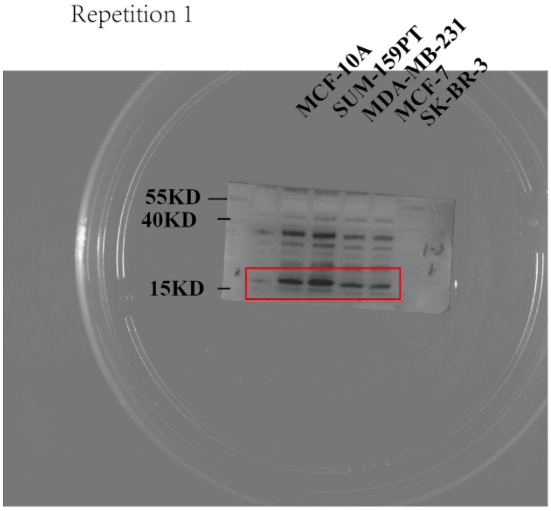

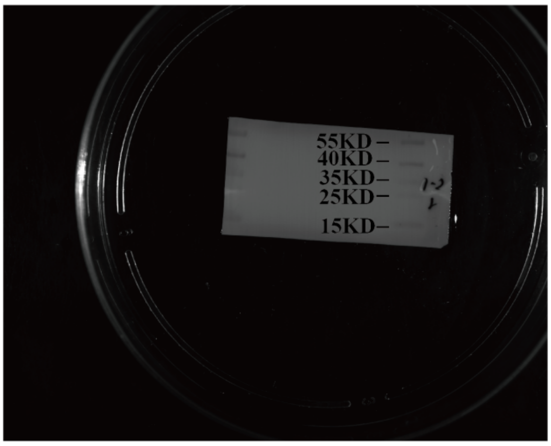

Repetition 2

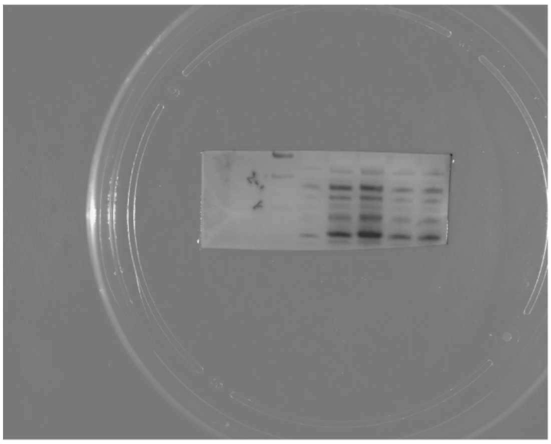

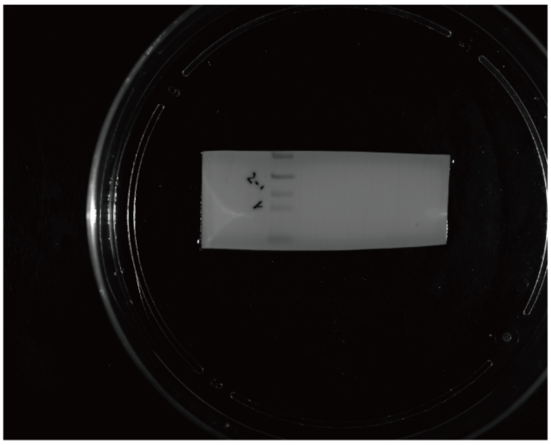

Repetition 3

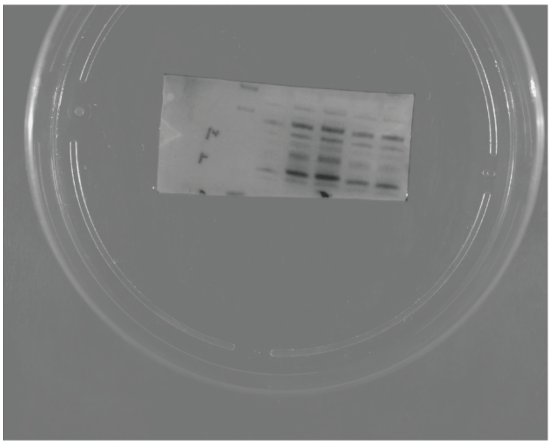

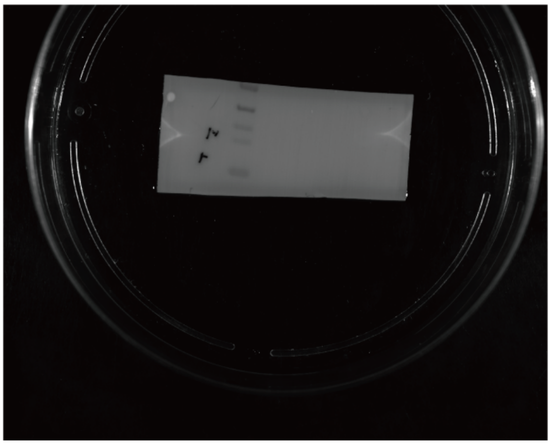

Sourse Data Fig.8B：CNR1: 66kDa

Repetition 1

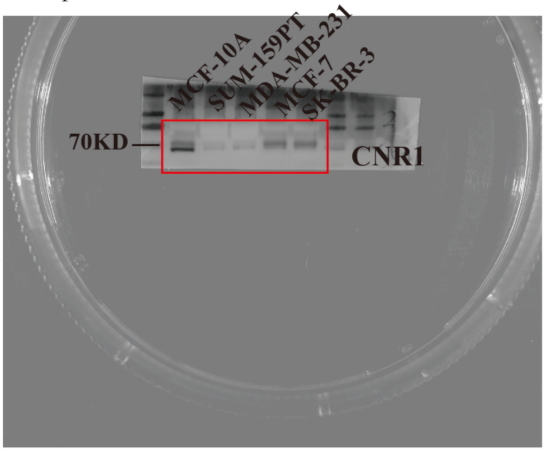

Repetition 2

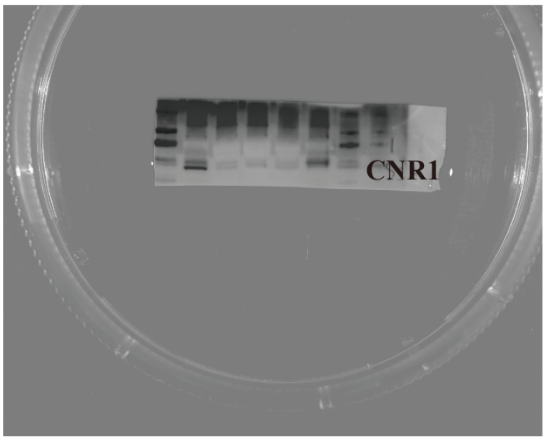

Repetition 3

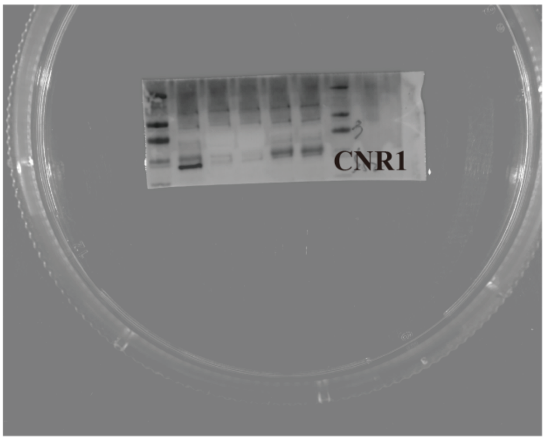

Supplement: Supplementary file 1 [file biology-15-01441-s001.zip › Supplementary Raw wb Image File.pdf]
